# Autonomic nervous system: an integrative regulator in circadian rhythm of blood pressure

**DOI:** 10.3389/fneur.2026.1766490

**Published:** 2026-06-29

**Authors:** Zhongyang Yu, Meng Zhao, Hongyue Xu, Ji Sun, Xiaoxing Jin

**Affiliations:** 1 State Key Laboratory of Cardiovascular Diseases, Shanghai East Hospital, School of Medicine, Tongji University, Shanghai, China; 2 Shanghai Frontiers Center of Nanocatalytic Medicine, Shanghai, China; 3 Department of Pathology and Pathophysiology, School of Medicine, Tongji University, Shanghai, China; 4 Clinical Center for Heart Disease Research, Tongji University, Shanghai, China; 5 Shanghai Arrhythmia Research Center, Shanghai East Hospital, School of Medicine, Tongji University, Shanghai, China; 6 Department of Cardiology, Shanghai East Hospital, School of Medicine, Tongji University, Shanghai, China; 7 State Key Laboratory for Diagnosis and Treatment of Severe Zoonotic Infectious Diseases, Key Laboratory for Zoonosis Research, Ministry of Education, Institute of Zoonosis, College of Veterinary Medicine, Jilin University, Changchun, China; 8 Department of Cardiology, The First Affiliated Hospital, Sun Yat-sen University, Guangazhou, Guangdong, China; 9 Department of Cardiology of Central China Fuwai Hospital, Central China Fuwai Hospital of Zhengzhou University, Zhengzhou, Henan, China; 10 Department of Cardiovascular Medicine, Fifth Affiliated Hospital of Sun Yat-sen University, Zhu Hai, China

**Keywords:** autonomic nervous system, blood pressure, circadian rhythm, heart, mechanism

## Abstract

Circadian rhythms, fundamental biological oscillations aligning physiological functions with the Earth’s 24-h cycle, profoundly influence blood pressure (BP) regulation. In this review, we first consolidates current understanding the systemic and cellular organizations of circadian clocks. Next, we delve into the intricate relationship between BP and circadian rhythms, examining how environmental cues like light, sleep, and diet disrupt these rhythms, leading to BP disorders. Crucially, we emphasize the ANS’s central role as a dynamic integrator, mediating the influence of central and peripheral circadian clocks on BP, and facilitating crosstalk with other major BP regulatory systems like the renin-angiotensin-aldosterone system (RAAS), natriuretic peptides (NP), and the immune system. Finally, we presented a future perspective of research on ANS function in the circadian rhythm of BP, identifying key unresolved questions and proposed therapies based on mechanisms for hypertension induced by ANS disorders. Overall, this is a comprehensive review elucidating ANS tone as a pivotal mechanism underlying the circadian rhythm of blood pressure.

## 1 Introduction

Circadian rhythms are fundamental biological oscillations. This profound influence on life, including cardiovascular regulation, is widely recognized. Blood pressure (BP) exhibits a distinct circadian rhythm, with the autonomic nervous system (ANS) tone considered a key factor in its regulation. Despite significant attention to the link between circadian rhythms and BP, existing literature often presents a fragmented view. Many reviews touch upon the ANS as a component, but a comprehensive synthesis focusing on ANS tone as a primary driver of BP circadian rhythm, particularly in the context of hypertension, is demanded for integrated understanding. How clock genes mechanistically modulate ANS tone throughout the day, and the effects of therapeutic strategies targeting the ANS, remain limited or scattered.

This review aims to bridge these conceptual and mechanistic gaps. We provide a comprehensive report that positions ANS tone as a crucial mechanism driving the circadian rhythm of BP. Our rationale is to consolidate current knowledge, highlight the ANS’s central role in both physiological BP rhythm and its pathological disruption in hypertension, and critically evaluate ANS-targeted diagnostic and therapeutic approaches. Specifically, we will elucidate the intricate mechanistic framework through which the ANS acts as a central mediator of cross-scale integration, integrating circadian signals from the suprachiasmatic nucleus (SCN) and orchestrating dynamic interactions with the RAAS, NP, and immune system to maintain BP homeostasis or contribute to hypertension. By doing so, we intend to offer a unified narrative that not only deepens the understanding of ANS function in BP chronobiology but also identifies promising avenues for future research and promising therapeutic interventions for hypertension.

## 2 Circadian clocks: systemic and cellular organization

### 2.1 Systemic organization of circadian clocks

**Figure 1:**
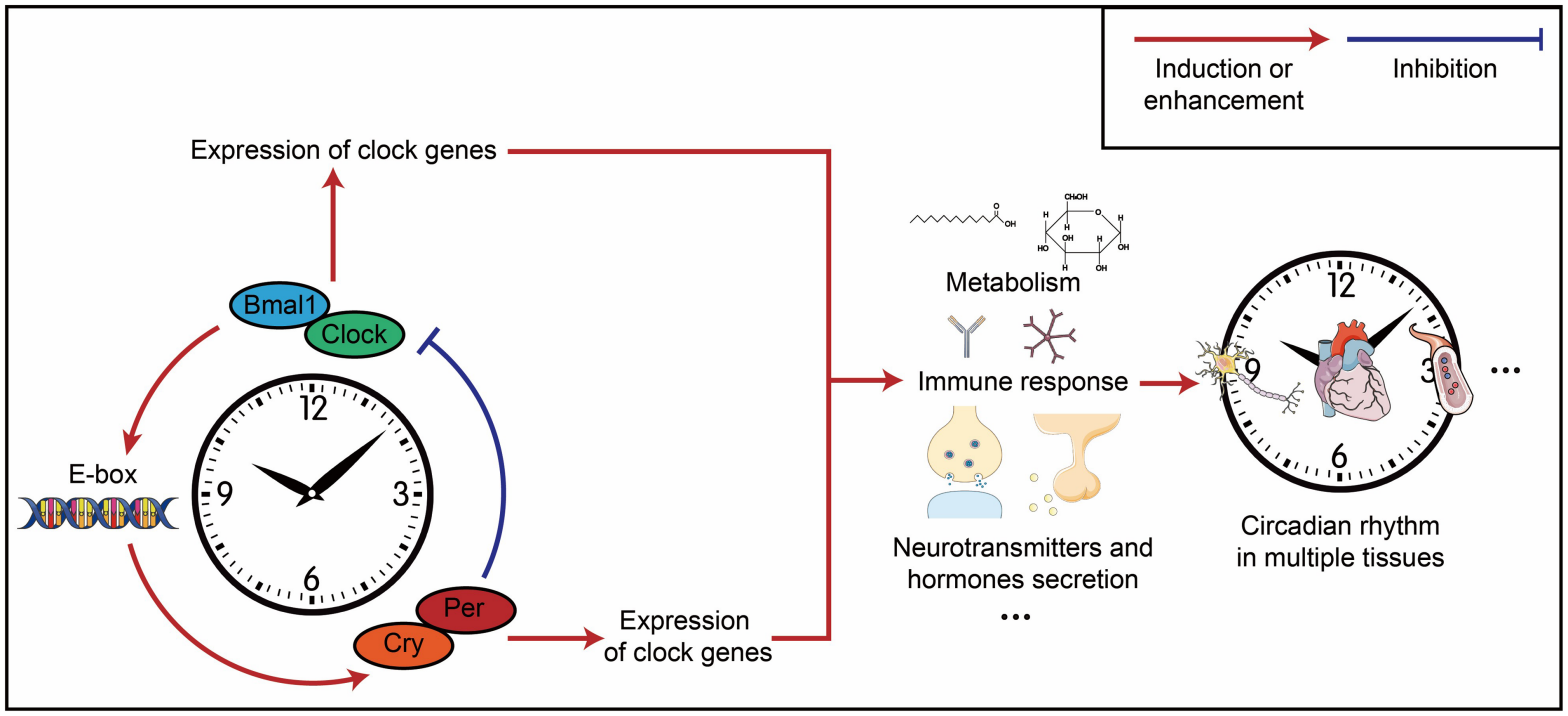
Systematic organization of circadian clocks and BP regulation under physiological conditions. This figure illustrates the hierarchical organization of circadian clocks. The suprachiasmatic nucleus (SCN), located in the hypothalamus, serves as the central clock, receiving external time cues (Zeitgebers) such as light (via the retina), sleep, and daily diet. The SCN then synchronizes peripheral clocks located in various organs (e.g., heart, kidneys) through both neural pathways [involving the autonomic nervous system (ANS)] and humoral pathways (e.g., hormonal signals from the pituitary gland). The ANS, comprising the sympathetic and parasympathetic systems, plays a crucial role in transmitting circadian signals to peripheral organs, thereby influencing blood pressure (BP) rhythms. Under physiological conditions, these synchronized clocks ensure that BP exhibits a characteristic diurnal variation (higher during the day, lower at night). This systematic regulation ensures the body’s physiological functions, including BP, are aligned with the earth’s 24-h day/night cycle.

Circadian rhythms are orchestrated by a hierarchical clock system. This system includes a central clock in the hypothalamus’s suprachiasmatic nucleus (SCN) and peripheral clocks in nearly every cell and organ (1, 2). External cues, primarily light, entrain the SCN, which then synchronizes peripheral clocks throughout the body via both neural and hormonal pathways (3–5). The ANS is a critical neural conduit in this process. It directly transmits circadian signals from the SCN to peripheral organs. Thus, the ANS acts as a vital bridge between central and peripheral clocks (Figure 1). Hormonal pathways, such as the hypothalamic–pituitary–adrenal (HPA) axis, also contribute to this synchronization. The ANS, acting as a key efferent pathway, transmits circadian signals from the SCN to peripheral targets, thereby playing a significant role in regulating physiological processes such as blood pressure (BP; Figure 1) (6, 7).

### 2.2 Cellular organization of circadian clocks

**Figure 2:**
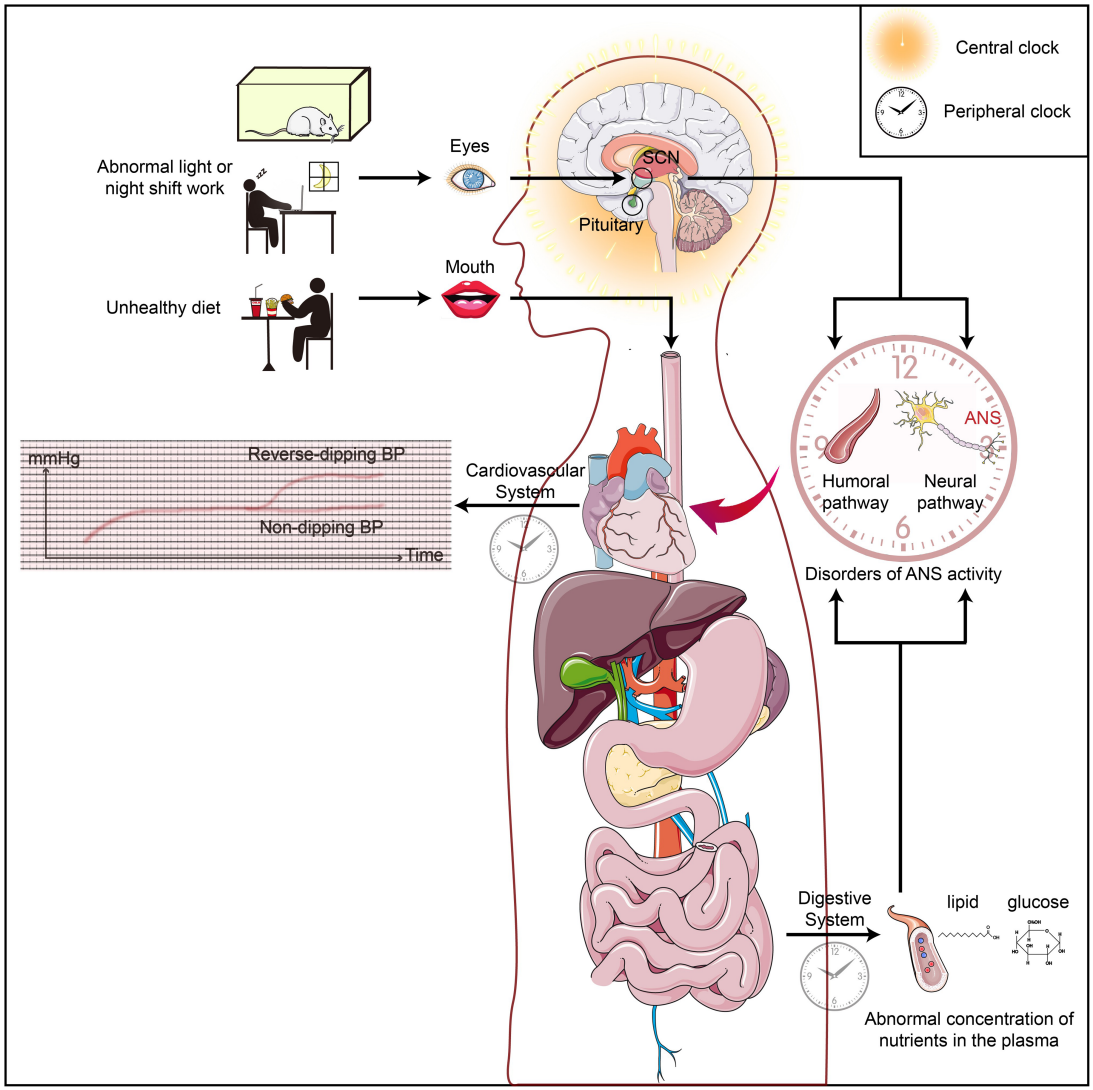
Cellular organization of circadian clocks and molecular regulation of BP. This figure illustrates the molecular mechanisms underlying circadian rhythms within individual cells, which constitute the peripheral clocks. At the core is the transcription-translation feedback loop (TTFL), a self-regulated system involving key clock proteins. Specifically, Clock and Bmal1 heterodimers (TFs) positively regulate the expression of Cry and Per genes at the beginning of a day. As Cry and Per proteins accumulate, they dimerize and subsequently inhibit the expression of Bmal1 and Clock. This cyclical inhibition and activation lead to the oscillation of these TFs throughout the day. These oscillating clock proteins then bind to E-boxes (enhancer box elements) in the promoters of clock-controlled genes (CCGs), thereby regulating their rhythmic expression. These CCGs are involved in diverse physiological processes, including metabolism (e.g., lipid, glucose regulation), immune response and the secretion of neurotransmitters and hormones. These molecular oscillations within peripheral clocks, particularly in organs like the heart and vessels, contribute to the circadian variation of blood pressure (BP). For instance, specific clock genes (e.g., Bmal1, Per1) have been shown to directly modulate BP rhythm and susceptibility to hypertension. This cellular organization ensures that cellular functions are synchronized with the daily cycle, ultimately impacting systemic physiological parameters like BP.

At the cellular level, circadian clocks are driven by a transcription-translation feedback loop (TTFL) (8, 9). These molecular oscillations regulate numerous clock-controlled genes. These genes are involved in diverse physiological processes, including metabolism and the secretion of neurotransmitters and hormones. These cellular clocks within ANS components and target organs are crucial for the rhythmic modulation of BP (Figure 2) (10). Critically, these molecular oscillations within peripheral clocks, particularly in cardiovascular tissues and those influencing ANS activity, contribute to the circadian variation of BP. Dysregulation of specific clock genes has been directly linked to altered BP rhythms and susceptibility to hypertension.

## 3 BP regulation by circadian rhythms

Circadian variation in BP is one of the earliest physiological phenomena of the cardiovascular system observed in humans. In diurnal humans, under physiological conditions, BP is greater during the daytime, typically exhibiting two peaks at 9 a.m. and 7 p.m. In contrast, BP is lower during the nighttime, with a maximal dip at 3 a.m. Under normal circumstances, the average BP of humans decreases by 10–20% at night, but the systolic BP (SBP) fluctuates more dramatically than the diastolic BP (DBP) (11).

However, some individuals with hypertension lose this reduction in BP at night. This type of abnormality is termed “nondipping.” Patients with nondipping BP account for a considerable proportion of hypertensive patients. Thus, in a way, hypertension can be regarded as a circadian rhythm disorder. Bianchi S et al. reported that patients with nondipping BP were at greater risk of left ventricular hypertrophy, cardiovascular events, cerebrovascular events and kidney injury, indicating that nondipping BP was more harmful to patients than dipping BP (12). The prevailing mechanistic hypothesis for nondipping BP centers on a dysregulation of the ANS and the renin-angiotensin-aldosterone system (RAAS). Specifically, nondipping is often associated with an attenuated nocturnal decrease, or even an increase, in sympathetic nervous system (SNS) activity, leading to sustained vasoconstriction and cardiac output during the rest phase. Concurrently, an impaired nocturnal suppression of RAAS activity, particularly aldosterone, can contribute to fluid retention and elevated BP during the night. Furthermore, defects in the central circadian clock (SCN) or its output pathways, as well as peripheral clock dysfunctions in organs like the kidney, are also implicated in disrupting the normal nocturnal BP decline (13). Notably, reverse-dipping BP is a manifestation of extreme circadian rhythm defects, which is especially worthy of a series of scientific studies (14, 15). This severe pattern, characterized by higher nocturnal BP than daytime BP, is often linked to profound ANS dysregulation, such as marked sympathetic hyperactivity during sleep, and can be exacerbated by conditions like obstructive sleep apnea syndrome (OSAS) or severe autonomic neuropathy (16). In these cases, the normal physiological shift toward parasympathetic dominance during sleep is severely blunted or reversed, leading to an inappropriate nocturnal increase in cardiovascular load.

At the molecular level, proteins encoded by clock genes in the central clock and peripheral clocks modulate multiple pathological phenotypes in both humans and rodents. Bmal1 is responsible for the onset of type 2 diabetes and hypertension by regulating the susceptibility of humans to these chronic diseases (17). In animal experiments, Bmal1 global knockout mice presented decreased mean BP (18), and Bmal1 knockout in smooth muscle cells impaired BP rhythm (19). The kidney is another organ that modulates BP. Marques et al. performed unbiased transcriptomic studies using kidney tissue from normotensive individuals compared with that from hypertensive individuals and reported that Per1 was significantly upregulated in the kidneys of hypertensive individuals compared with those of normotensive individuals (20). In addition, Stow et al. reported that Per1 modulated renal sodium transport and thereby influenced BP (21). In the adrenal gland of mice, Clock mutations dampened the diurnal rhythm of arterial pressure and heart rate compared with those of normal mice (22). Furthermore, Cry1/2 deficiency in mice increased susceptibility to salt-sensitive hypertension due to the abnormal concentration of the mineralocorticoid aldosterone (23). In summary, BP is higher in the daytime and lower at night, and clock genes in peripheral clocks modulate BP in terms of molecules (Figure 2). While these molecular mechanisms show conservation across species, the precise timing and physiological manifestation of their effects on BP rhythms must be interpreted in the context of the species’ chronotype.

## 4 BP disorders induced by disrupted circadian time cues

The circadian rhythm of BP in our bodies is affected by environmental cues. As a result of the rotation of the Earth, light is the main cue used to regulate BP. Moreover, an appropriate duration and high quality of sleep can eliminate the adverse effects of a disrupted day/night cycle. In addition to light and sleep, daily diet is another main cue for BP, and the timing and content of feeding regulate the concentration of nutrients in our bodies in a circadian pattern and thereby influence BP (Table 1).

**Table 1:** Circadian cues and BP alterations mediated by different mechanisms.

Circadian cues		Animal models or clinical participants	Mechanisms		Alterations of blood pressure (BP) circadian rhythm	Reference
Light or shift work	12:12 light/dark cycle (L/D), constant dark (D/D) and constant light (L/L)	Smooth-muscle–specific Bmal1-knockout mice (SM-Bmal1–KO)	ANS	SM-Bmal1–KO deletion abolished the time-of-day variations in response to agonist-induced vasoconstriction, myosin phosphorylation, and ROCK2 activation	The amplitude of BP fluctuation was decreased in the L/L group and D/D group compared with the L/D group, and constant light significantly increased acrophase and period length	Xie et al.
	L/L and L/D	Superior cervical ganglionectomy (SCGx) and 6-hydroxydopamine sympathectomy (SYMPx)	ANS	Light-induced increase in sympathetic outflow could suppress the circadian rhythm of BP	SCGx and SYMPx completely abolished the circadian rhythm of BP	Briaud et al.
	Artificial light at night (ALAN) and L/D	Normal adult male Wistar rats	ANS and RASS	ALAN decreased low-frequency bands (a sympathetic marker)	ALAN suppressed 24-h variability of pulse pressure in the rat thoracic aorta	Mauer et al.
	L/L and L/D	Normal adult male Wistar rats	ANS	L/L increased recording renal sympathetic nerve activity (RSNA) and plasma level of norepinephrine (NE)	Circadian disruption increased BP	Duan et al.
	Dim light at night (dLAN) and L/D	Normal adult male Wistar rats	ANS	In female mice, dLAN decreased the relative sympathetic regulation at night, while in male mice it increased the relative sympathetic regulation during the daytime	In female mice, dLAN reduced the amplitude of day-night BP at night, and in male mice, it increased the amplitude of BP during the daytime.	Prabhat et al.
	Shift work	European white men	Inflammation	Sleep length influence the inflammatory status	Poor sleep quality and permanent night shift work are positively associated with systolic BP/diastolic BP	Kanki et al.
	Shift work	Healthy adults	ANS and inflammation	Circadian misalignment decreased wake cardiac vagal modulation and increased 24-h serum interleukin-6, C-reactive protein, resistin, and tumor necrosis factor-α levels	Circadian misalignment increased 24-h systolic BP and diastolic BP by 3.0 mmHg and 1.5 mmHg	Morris et al.
	Shift work	Male shift workers	ANS	Shift work decreased parasympathetic modulation	Shift work increase blood pressure in a day	Souza et al.
	Shift work	Chronic shift workers	Inflammation	Circadian misalignment increased hs-CRP and blood pressure in shift workers	Circadian misalignment increased 24-h systolic BP and diastolic BP by 1.4 mmHg and 0.8 mmHg	Morris et al.
	Shift work	Physician completing a 24 h on-call duty (OCD) and a 24 h control period including a regular 8 h non-OCD.	ANS	Urinary noradrenaline excretion was greater during OCD when compared with control day	After OCD, diastolic BP throughout 24 h and in the night-time increased, and systolic BP during sleep time increased	Rauchenzauner et al.
Diet	Light-phase time-restricted feeding (TRF)	Diabetic mice (db/db mice) & mPer2Luc mice	ANS	TRF increased urinary NE and Epi during the light phase, and decreased urinary NE and Epi during the dark phase	Increased mean aterial pressure (MAP) during the light phase, and decreased MAP during the dark phase after TRF in db/db mice	Hou et al.
	Early time-restricted feeding (eTRF)	Men with Prediabetes	Concentration of glucose in the plasma	eTRF increased insulin sensitivity	eTRF lowered morning levels of systolic and diastolic BP	Sutton et al.
	TRF	Participants with metabolic syndrome and healthy participants	Concentration of glucose in the plasma	eTRF increased insulin sensitivity	TRF intervention impoved the effects of anti-hypertensive drugs	Wilkinson et al.
	TRF	Shift workers	Concentration of glucose in the plasma	TRF reduced glycated hemoglobin A1C	TRF reduced BP in a day	Manoogian et al.
	Sodium restriction diet	Patients with essential hypertension	Concentration of sodium in the plasma	Sodium restriction diet reduced nocturnal BP in sodium-sensitive subjects	Sodium restriction shifted circadian rhythm of blood pressure from nondipper to dipper in essential hypertension	Uzu et al.
	High fat diets	Sprague–Dawley rats (SD)	RASS	High fat diets activate RASS in female SD rats	High fat diets induced hypertension in female SD rats	Tain et al.

### 4.1 Light and sleep

The light on the Earth oscillates during the day/night cycle because of rotation. As a result, many physiological indices are synchronized with light fluctuations. For example, heart rate varies, as it has a relatively high frequency during the daytime and decreases when individuals fall asleep (24).

**Figure 3:**
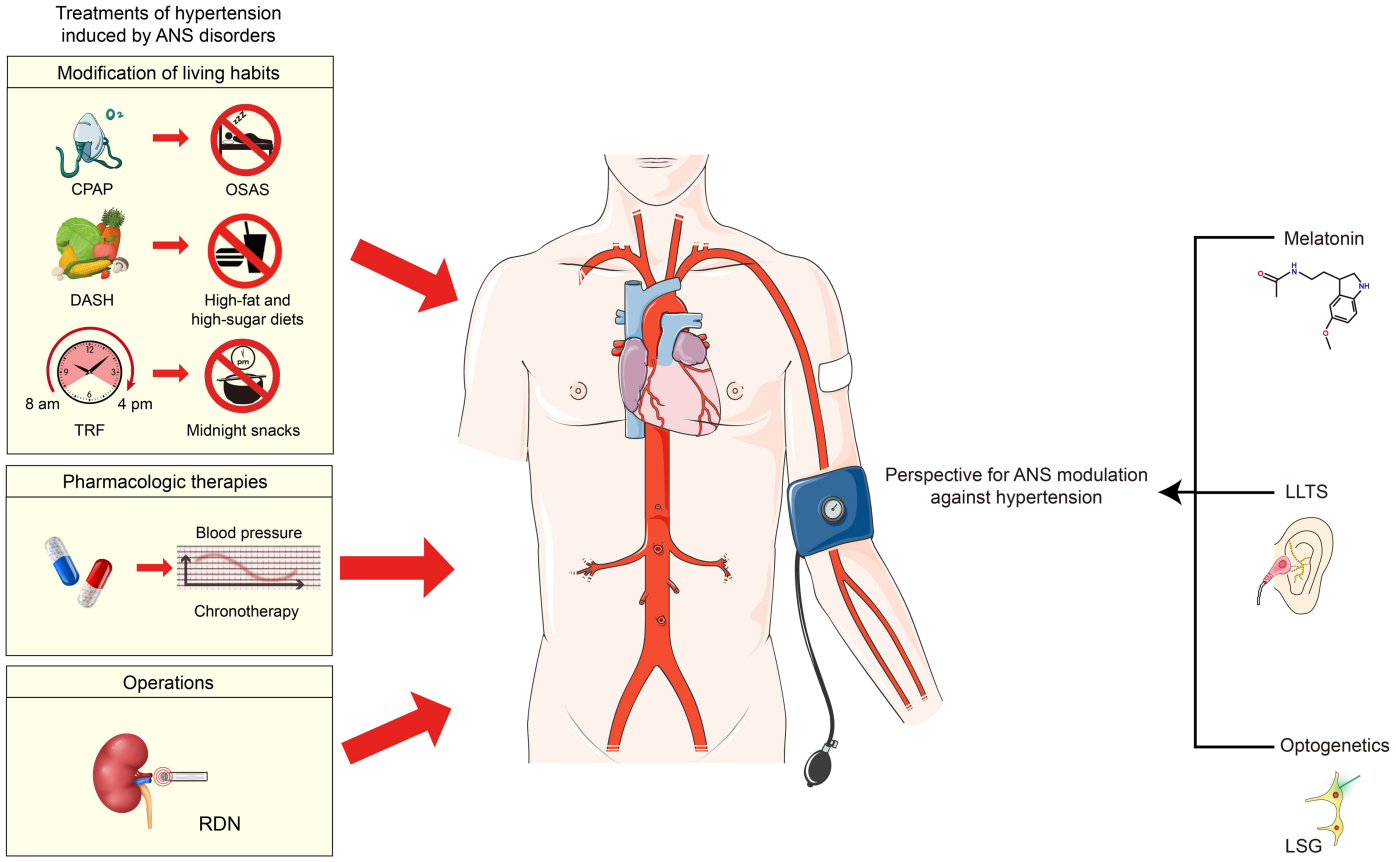
Disruption of circadian clocks and autonomic nervous system (ANS) activity by environmental cues in pathological conditions. This figure illustrates how various environmental disruptions initiate the pathological cascade leading to abnormal blood pressure (BP) patterns. External circadian time cues, such as abnormal light exposure or night shift work (affecting the natural light–dark cycle and sleep patterns) and unhealthy diet (e.g., high-salt, high-fat, or mistimed feeding), act as primary disruptors. These cues directly or indirectly impact the suprachiasmatic nucleus (SCN), the central clock, and subsequently perturb the synchronization of peripheral clocks located in various organs throughout the body. A critical consequence of these disrupted circadian signals is the disorder of ANS activity. This typically involves an imbalance between the sympathetic and parasympathetic nervous systems, often characterized by increased sympathetic tone and/or reduced parasympathetic modulation. This ANS dysregulation is a central mediator linking environmental disruptions to the development of hypertension. The overall effect is a deviation from the normal physiological BP rhythm, setting the stage for conditions like non-dipping and reverse-dipping BP. This figure highlights systemic impact of disrupted cues on the body’s timekeeping and regulatory systems.

BP is also notably affected by light intensity. Mice are a classic experimental model in circadian rhythm research. For these rodents, which are nocturnal, “day” represents their rest phase, whereas “night” corresponds to their active phase. To describe the influence of light on BP fluctuations, Xie et al. divided wild-type mice into three conditions, namely, 12:12 light/dark (L/D), constant dark (D/D), and constant light (L/L), and reported that the amplitude of BP fluctuations was lower in the L/L and D/D groups than in the L/D group and that constant light significantly increased acrophase and period length. Moreover, clock genes were found to participate in the modulation of circadian BP by light. Compared with wild-type mice, Per2 mutant mice lost their BP rhythm under L/D and D/D conditions (25). Other clinical trials have focused on the relationship between hypertension and circadian misalignment, which is common for real-life shift workers. A study built a model of circadian misalignment by programmed illumination, which was the opposite of natural light, and the results revealed that SBP increased 3.0 mmHg (5.6 mmHg during the sleep period and 1.6 mmHg during the wake period) and that DBP increased 1.5 mmHg (1.9 mmHg during the sleep period and 1.4 mmHg during the wake period) in the circadian misalignment group (Figure 3) (26, 27). These studies used highly sensitive, validated ambulatory BP monitoring (ABPM) devices capable of detecting subtle acute changes. Furthermore, it highlights that such sustained, albeit modest, elevations—when accumulated over prolonged periods as seen in shift workers—are recognized to contribute to the cumulative risk of chronic hypertension and cardiovascular events.

The above studies presented the effects of light on BP from the perspective of distinct light durations. In some studies, researchers have investigated how “dim light” or “artificial light” at night (ALAN) affects BP and reported that ALAN can diminish SBP variability accompanied by changes in inflammatory factors and metabolites, which also highlights the harmfulness of light disorders (28–31). Thus, light is a dominant circadian time cue for the oscillation of BP, and its disturbance has been associated with adverse effects on BP. Certainly, while ALAN is harmful, the precise dose–response relationship and long-term human clinical outcomes remain areas of active research. A significant methodological limitation in many ALAN studies is the reliance on animal models, which, despite providing mechanistic insights, may not fully capture the complex interplay of human behavioral, social, and psychological factors influencing sleep and BP regulation in response to light. Furthermore, human studies on ALAN often face challenges in controlling for confounding variables such as sleep hygiene, stress levels, and pre-existing cardiovascular conditions.

Sleep is not only a passive behavior regulated by natural factors, including day/night cycles but also an initiative behavior associated with social factors, including night shifts (32). Many scientists have reported that sleep disorders are correlated with BP disorders, especially with nondipping hypertension (33–35). Among all sleep disorders, scientists have focused particularly on OSAS (36). Patients with OSAS often have difficulty breathing because of nasopharyngeal stenosis; therefore, they always snore loudly (37). OSAS is prevalent in hypertensive patients, and the severity of hypertension is strongly related to the occurrence of apnea and oxygen saturation during sleep (38–40). The mechanisms by which hypertension is associated with OSAS are described below (Figure 3).

### 4.2 Daily diet

Feeding is another basic behavior of all animals. Since the SCN is the central clock in animals, it also regulates metabolic homeostasis by modulating food intake. The concentrations of multiple metabolites, such as glucose (41), lipids (42) and amino acids (43), oscillate with the pace of feeding. These metabolites play important roles in the progression of obesity and diabetes (44), which contribute to cardiovascular diseases (CVDs), including abnormal BP (45, 46).

Many studies have shown that unhealthy dietary habits, such as a high-fat diet (HFD), are associated with hypertension. Erdos et al. demonstrated that mice fed a HFD had elevated mean arterial BP (MABP), DBP and SBP (47). Carroll et al. reported that BP variability between day and night was increased in obese rabbits fed corn oil and lard (48). These studies support the idea that a HFD may lead to hypertension. While these animal models provide strong mechanistic evidence for the role of HFD in hypertension development, it is important to note that human studies, while generally supporting a link between high-fat intake and cardiovascular risk, often show a more complex relationship with BP regulation, influenced by overall dietary patterns, genetic predispositions, and other lifestyle factors (49). The direct translation of specific HFD-induced BP changes observed in rodents to human clinical outcomes requires careful consideration. This is largely due to fundamental differences in metabolism, dietary composition, and the complexity of human eating patterns compared to controlled animal feeding experiments. A key knowledge gap lies in understanding the precise molecular and neural pathways through which various dietary components, beyond just fat and salt, interact with the ANS and circadian clocks to influence BP in diverse human populations.

Moreover, salt in food also affects BP changes caused by plasma osmotic pressure. Most patients with nondipping hypertension are sensitive to salt (50). In the Dahl salt-sensitive rat model, the MAP in the high-salt diet group was much greater than that in the normal-salt diet group and maintained the amplitude even after the rats were returned to a normal-salt diet, indicating that salt-sensitive hypertension may be irreversible and that the harmfulness of a high-salt diet should not be ignored (Figure 3) (51). The findings from salt-sensitive animal models, such as the Dahl rat, have been instrumental in understanding the genetic and physiological basis of salt-sensitive hypertension. These insights are highly relevant to humans, as a significant proportion of hypertensive individuals exhibit salt sensitivity, where high dietary sodium intake directly contributes to elevated BP and increased cardiovascular risk (52). However, the degree of salt sensitivity varies widely among human populations, influenced by ethnicity, age, and underlying health conditions, suggesting that while the mechanism is conserved, its manifestation can differ. This variability presents a methodological challenge in clinical trials, as a “one-size-fits-all” approach to salt restriction may not be equally effective across all individuals. Further research is needed to develop personalized dietary recommendations based on individual salt sensitivity and genetic profiles.

## 5 Mechanisms behind circadian variation in BP

BP is a widely used parameter in clinical practice and is associated with a high risk of CVDs. Nevertheless, scientists have not elucidated the entire mechanism of BP regulation because it is a result of complicated interactions between environmental and pathophysiological factors and usually acts on a genetic background. Here, we list several reported main endogenous regulators of BP, particularly the ANS.

### 5.1 ANS

#### 5.1.1 Origin and characteristics of ANS tone

The ANS is a pivotal regulator of BP, comprising the SNS and the parasympathetic nervous system (PNS). Both exhibit distinct circadian patterns, with SNS activity predominating during wakefulness and PNS activity during sleep, as evidenced by heart rate variability (HRV) (53). The evidence presented by Han et al. about sympathetic ganglions describes circadian patterns of the ANS in a direct way. In the acute myocardial ischemia (AMI) model of canines, they implanted equipment to record the activity of the ANS directly and reported that the activity of SNS discharges has remarkable circadian variation, but there is no evidence for nocturnal elevation of absolute vagal discharges (54). Ogawa et al. drew a similar conclusion in the pacing-induced congestive heart failure model of canines; that is, they argued that the relative predominance of vagal tone at night was the result of reduced SNS activity without an absolute increase in vagal discharges (55). These studies help us understand the origin of the ANS tone and explore its potential mechanisms.

As mentioned above, the ANS is necessary when the SCN synchronizes peripheral clocks such as the heart; therefore, the ANS tone clearly fluctuates under the influence of the SCN during the day. The oscillation of light in the environment entrains the SCN by influencing neuronal activity in the nucleus. As it is located in the hypothalamus, the SCN has wide-ranging connections with other nearby regions to modulate basic behavioral functions such as food and water intake, sleep and reproduction. The paraventricular nucleus (PVN) is the critical nucleus in the regulation of SNS activity. The SCN projects neuron fibers to the PVN and synchronizes with it and other nuclei, such as the rostral ventromedial medulla. During the daytime, light activates the SCN, transmitting the message into the PVN, increasing the SNS tone. The subsequent nocturnal reduction in the SCN leads to a decrease in SNS tone. These are explanations for ANS tone changes throughout the day (Figure 1) (56). However, the regulation of BP by this axis is not a simple linear cascade but involves intricate bidirectional feedback loops. For instance, afferent signals from baroreceptors, sensing changes in BP, feed back to the brainstem and subsequently influence PVN activity, modulating SNS output. Moreover, neurohumoral factors, such as circulating catecholamines and angiotensin II (as discussed in Section 5.2), can directly influence both central (e.g., PVN) and peripheral components of the ANS, creating complex regulatory networks that extend beyond direct neural projections. This neurohumoral crosstalk is crucial, as ANS activity can modulate the release and sensitivity of these factors, while they, in turn, can feedback to alter ANS tone, forming a tightly regulated circadian network. Furthermore, while the SCN acts as the master clock, peripheral clocks located in various organs (e.g., adrenal glands, kidneys, and vasculature) also play a significant role in local ANS activity and BP control. These peripheral clocks, though entrained by the SCN, can be independently influenced by local environmental cues and metabolic signals, and their dysregulation can contribute to BP abnormalities, potentially feeding back to influence central ANS regulation.

#### 5.1.2 BP modulation by ANS activity

The amplitude of BP is related to multiple factors, including blood volume, cardiac output and vascular resistance (57). During the regulation of BP, the crosstalk between the ANS and physicochemical receptors such as baroreceptors bridged with the afferent nerves and the efferent nerves (58, 59). When BP decreases baroreceptors input signals through the afferent nerves in the ANS to the brain, and the brain subsequently integrates the signals and increases the activity of efferent nerves in the SNS (57, 60). After the activation of SNS, catecholamine was secreted and regulates BP (61). PNS modulates BP via similar mechanisms but with opposite results (62). Above all, ANS activity influences BP in these ways.

ANS plays a crucial role in the progression of hypertension, accompanied by increased SNS activity and reduced PNS activity (63, 64). In clinical practice, the levels of norepinephrine and epinephrine in plasma and urinary catecholamine concentrations usually serve as indicators of SNS activity. However, it is important to note that plasma catecholamine levels are influenced by several factors beyond direct SNS outflow, including their rapid clearance from circulation, the precise timing of blood sampling relative to physiological events, and the patient’s acute stress response during sampling. Therefore, while valuable, these measurements provide an indirect assessment of SNS activity and require careful interpretation (65). The concentration of catecholamines in patients’ plasma has revealed that many normotensive individuals with a family history of hypertension exhibit abnormal SNS discharge activity (66). High levels of catecholamine were also found in people with hypertension after matching for age (67). In experiments of animals, SNS has been shown to be involved in various types of pathological processes in hypertension. Furthermore, SNS activation promotes the absorption of sodium and thereby maintains a high level of BP. Fujita et al. reported that *α*-1 adrenergic receptor downstream pathways are associated with dysfunction of endothelial cells and disorders of vasoconstriction, which is another pattern of SNS cross-talk with hypertension (68). These studies prove that an imbalance in the ANS plays an important role in the generation and progression of hypertension (Figure 3).

#### 5.1.3 Circadian rhythm of BP oscillations with ANS tone over 24 h

The above studies proved that the ANS is a dominant component of BP regulation and that the circadian rhythm of BP is influenced by ANS tone changes over 24 h. Specifically, under physiological conditions, when SNS activity increases, contraction of the myocardium and smooth muscles in vessels increases, and the heart beats rapidly, resulting in a morning surge in BP. The PNS works in the opposite way to the SNS. Under pathological conditions, abnormal ANS tone over 24 h is the major cause of the onset and progression of hypertension. Animal experiments have shown that constant light can activate SNSs and increase BP during the day (69), and sympathectomy inhibits the influence of SNSs on BP (70). Constant light in nocturnal animals activates the SNS and raises BP during rest, disrupting normal nocturnal elevation. While these models clarify light’s direct impact, their relevance to humans—where light exposure is complex and rarely constant—requires caution. Furthermore, as sympathectomy is an extreme intervention, its findings indicate SNS involvement (proof-of-concept) rather than serving as a direct therapeutic strategy. In contrast to its profound physiological impact, the imbalance of the ANS is often underdiagnosed or not routinely assessed in general clinical practice, despite its clear manifestations in specific patient populations. Night shift workers showed decreased parasympathetic modulation and increased BP (71). Physicians suffering from 24-h on-call duty (OCD) had higher urinary noradrenaline excretion and DBP throughout 24 h, and at night, these values increased (72). While these observational studies strongly suggest a link between disrupted circadian rhythms and altered ANS activity leading to elevated BP, it is crucial to acknowledge that populations like night shift workers and physicians on-call are often simultaneously exposed to multiple confounding factors. These include, but are not limited to, chronic sleep deprivation, increased psychological stress, and potentially less healthy lifestyle choices (e.g., irregular eating patterns, reduced physical activity) (73). These factors can independently, or in conjunction with circadian misalignment, contribute to ANS dysregulation and hypertension. Patients with hypertension secondary to diabetes also presented with BP disorders. Physicians have reported that dysautonomia in diabetic patients eliminates the circadian variation in BP (74, 75). Fatal familial insomnia (FFI), a prion disease with broken sleep–wake cycles, also shows a nondipping BP pattern and is characterized by progressive alterations in neurohormonal and cardiovascular rhythms, indicating that the nondipping BP pattern in these patients is due to deficits in pituitary–adrenal function (76). Patients with OSA also exhibit strong sympathetic activity. When they are in hypoxia, the oxygen-conserving reflex induced by chemoreceptors leads to vasocontraction mediated by sympathetic hyperactivity to ensure the blood flow to vital organs, causing an imbalance in the ANS and promoting the progression of hypertension (77, 78).

As mentioned above, unhealthy dietary habits are also responsible for hypertension. In fact, as the prime motivator for hypertension, a high-salt diet can disturb the circadian rhythms of plasma sodium, activate the SNS and thus mediate the adverse effects on BP in spontaneously hypertensive rats (79, 80). In patients with essential hypertension, clinicians have also reported the harmful influence of a high-salt diet on morning BP, and a high-salt diet induces increased SNS activity and elevated plasma noradrenaline concentrations in patients (81). Moreover, the nuclei associated with the SNS in the CNS also participate in the process of circadian BP regulation induced by a high-salt diet (82–84). Hence, a high-salt diet stimulates SNSs directly and indirectly. Patients with diabetes are at high risk of hypertension, and the onset of diabetes is usually related to an unhealthy diet (85). Farah et al. explored the influence of fructose on BP and the SNS in diabetes (86), and they reported that excessively consuming fructose could increase nocturnal BP and aggravate ANS imbalance by activating the SNS. These results suggest that patients with diabetes should pay more attention to their daily diets, especially the consumption of fructose. While animal studies consistently demonstrate that high fructose intake can induce hypertension and ANS dysregulation, human epidemiological and interventional studies also support a link between excessive fructose consumption and increased risk of hypertension, metabolic syndrome, and CVDs (87). However, the magnitude of effect and the specific mechanisms may differ between species, and human dietary patterns are often more complex than isolated fructose intake. In summary, light and diet are the main circadian cues for BP regulation, and ANS disorders induced by disrupted cues significantly increase BP and even lead to the onset of hypertension.

### 5.2 RASS

The RASS has a wide effect on the regulation of BP. Renin, a type of acid proteinase synthesized and secreted by juxtaglomerular cells, cleaves angiotensinogen into angiotensin I (Ang I), which is then cleaved into Ang II by angiotensin-converting enzyme (ACE). Ang II can increase BP via a vasoconstrictive effect and critically, activates the ANS by binding to AT receptors, thereby enhancing sympathetic outflow and contributing to vasoconstriction and increased cardiac output. This direct activation establishes a significant feed-forward loop between RAAS and ANS, where increased Ang II amplifies sympathetic activity, which in turn can stimulate renin release, perpetuating the cycle (57, 61). Ang II also induces aldosterone synthesis and release to increase the reabsorption of sodium.

RASS plays an important role in the occurrence of primary and secondary hypertension. In patients with IgA nephropathy, the circadian rhythm of BP is significantly altered by angiotensinogen (88). RASS also shows an obvious pattern of circadian rhythm. It is more active in the morning than in the afternoon, which could serve as a potential mechanism for fluctuations in physiological and pathological BP (89, 90). Increased Ang II leads to endothelial dysfunction and an inflammatory response accompanied by promoted oxidative stress, which contributes to extensive injury to vessels (91). Abnormal concentrations of RASS molecules not only in the circulation but also in the kidneys can lead to high BP. Higher BP at night is related to the circadian disorders of intrarenal RASS followed by renal dysfunction (92). A study in rats showed that HFD induced hypertension via RASS activation (93). Thus, RASS dominates the blood volume and is critical for the modulation of BP.

### 5.3 Natriuretic peptide

Atrial natriuretic peptide (ANP) and brain natriuretic peptide (BNP) are hot topics because of their positive effects on the regulation of BP. When the atrium and ventricle are stretched, they release ANP and BNP into the blood circulation and decrease the blood volume. NP can increase efferent arteriolar tone in the kidneys and thereby increase the glomerular filtration rate. NP inhibits renal sodium reabsorption by decreasing the activity of Na^+^/K^+^-ATPase and the sodium–glucose cotransporter in the proximal tubule (94).

In 1995, scientists discovered that concentrations of long-acting NP and sodium Fannie factor (ANF) have a circadian rhythm: higher during sleep, and then reduced to half that in the afternoon and evening. SBP, DBP and MABP are also rhythmic, but they have opposite wave peaks and wave troughs to those of long-acting NP and ANF. These results highlighted the importance of the modulation of BP by NP and ANF (95). The circadian rhythm of NP secretion is influenced by various factors, including atrial stretch, which itself can be modulated by ANS activity. For instance, sympathetic activation can alter cardiac contractility and preload, indirectly affecting NP release, while NP can exert counter-regulatory effects on sympathetic tone by inhibiting norepinephrine release and reducing sympathetic nerve activity, thus forming a crucial negative feedback loop in BP regulation (96). In patients with heart failure, high doses of NP can also inhibit renal sympathetic nerve activity (97). In a cross-sectional trial, Parcha et al. screened 40 participants, 18 lean and 22 obese, and reported that obesity could lead to the loss of the NP rhythm and further disturb fluctuations in BP (98). Similarly, Tamura et al. observed that NP level influenced nocturnal BP when there was aortic stenosis (99). These studies proved that the rhythm of the NP influences the oscillation of BP.

### 5.4 Inflammation and the immune system

The immune system has been demonstrated to be related to the development of hypertension. Inflammation in the vascular wall involves many immune cells and immune molecules. With the release of powerful mediators, the reduction in lumen diameter and the promotion of vascular fibrosis, vascular permeability and vascular resistance increase (100). For example, the NLRP3 inflammasome can significantly promote the formation of cholesterol crystals, which are the main pathological phenomenon in atherosclerosis (101). Therefore, the NLRP3 inflammasome may be involved in exacerbating hypertension. Similarly, the deletion of CXCL3 and CCR2 was also associated with the course of atherosclerosis (102). Crucially, the autonomous nervous system plays a significant role in modulating immune responses and inflammation through the neuroimmune axis. Sympathetic activation can promote pro-inflammatory states, while parasympathetic activity, particularly via the vagus nerve, can exert anti-inflammatory effects. Neurotransmitters such as norepinephrine released by splenic sympathetic nerves can activate splenic T cells and contribute to hypertension (103). In turn, inflammatory factors can stimulate SNS and promote hypertension (104). With respect to circadian rhythms, Boos et al. proposed that the neutrophil-lymphocyte ratio (NLR) was correlated with the ambulatory arterial stiffness index (AASI), which is an index obtained from ABPM and is associated with reverse dipping and nondipping hypertension (105). Kanki et al. reported that poor sleep quality and permanent night shift work are positively associated with C-reactive proteins, which might be the reasons for increased SBP and DBP (106). These findings underscore the complex interplay where circadian disruption, often mediated by altered ANS tone, can drive inflammatory processes that contribute to hypertension. In conclusion, inflammation and the immune system are closely related to hypertension because they promote various pathological processes, and their circadian regulation is intricately linked to ANS activity.

## 6 Diagnosis and treatments for hypertension induced by ANS disorders

Hypertension is the most important risk factor for all-cause morbidity and mortality worldwide and is associated with an increased risk of CVDs (107). However, more than half of patients with hypertension do not receive sufficient treatment to control the illness, and screening for nondripping hypertension is needed. Therefore, early diagnosis and intervention of hypertension are valuable and need improvement. Given the critical role of ANS disorders in the progression of hypertension, approaches involving the ANS should also be integrated during diagnosis and intervention.

### 6.1 Diagnosis of hypertension induced by ANS disorders

**Figure 4:**
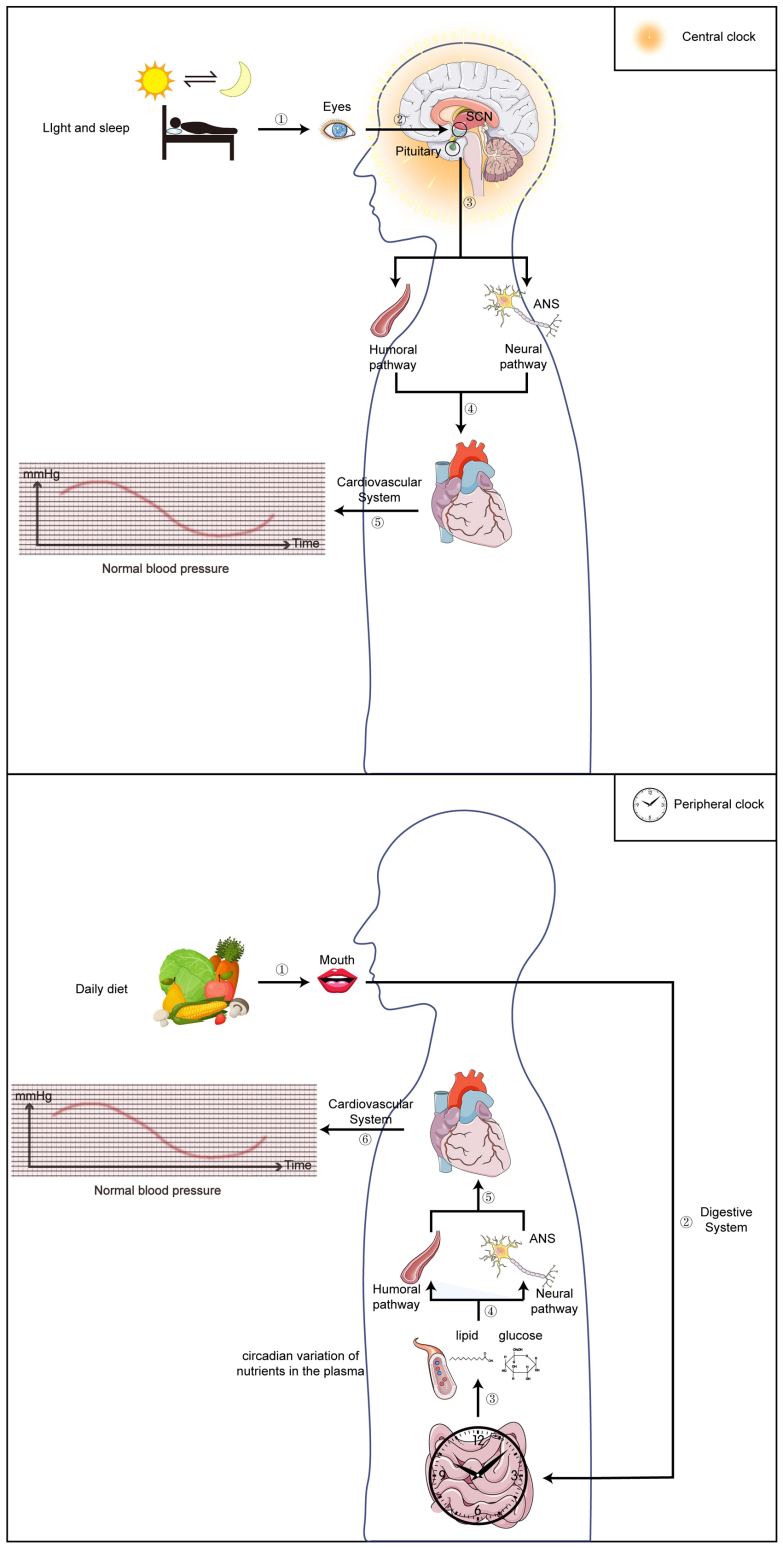
Therapeutic strategies modulating the autonomic nervous system (ANS) for hypertension. This figure outlines the therapeutic strategies for hypertension through modulation of the autonomic nervous system (ANS), summarizing the mechanisms of current and prospective treatments. These strategies are categorized into four main groups: first, lifestyle modifications, which encompass treating obstructive sleep apnea syndrome (OSAS) with continuous positive airway pressure (CPAP) to rebalance the ANS and improve blood pressure (BP) control, adopting the Dietary Approaches to Stop Hypertension (DASH) diet rich in fruits/vegetables and low in saturated fats to modulate the intestinal sympathetic nervous system, and employing time-restricted feeding (TRF) to inhibit sympathetic activity and enhance insulin sensitivity; second, pharmacological chronotherapy, which optimizes the timing of antihypertensive medication administration (e.g., before sleep) to target circadian BP fluctuations and ANS activity for enhanced efficacy; third, interventional treatment via catheter-based renal denervation (RDN), a minimally invasive procedure that ablates renal sympathetic nerves to reduce SNS activity and lower BP, demonstrating significant results especially in resistant hypertension; and finally, emerging perspectives involving melatonin therapy to rebalance the ANS by increasing parasympathetic and attenuating sympathetic activity (particularly in non-dipping hypertension), low-level tragus stimulation (LLTS) to correct ANS dysregulation, and optogenetics for precise, light-mediated control of neuronal activity (e.g., targeting sympathetic ganglia such as the LSG), offering a highly specific non-pharmacological therapeutic avenue.

ABPM is the main assay used to monitor BP within 24 h (108). The application of ABPM can provide precise information about oscillations of BP in a day (109–111). ABPM can also screen patients with white-coat hypertension and masked hypertension (112). Therefore, its use provides convenience for diagnosing and tapping potential patients with primary hypertension, whereas ANS disorders are the main reason for hypertension, as mentioned above; thus, ABPM is often combined with monitoring of the ANS to explore whether hypertensive patients have ANS disorders. To determine the correlation between muscle sympathetic nerve activity (MSNA) and day–night BP changes, scientists have inserted microelectrodes into the peroneal nerve in hypertensive patients and concluded that MSNA may be responsible for the day–night BP variation (113–115). There are also several noninvasive approaches to assess sympathetic nerve activity, such as the concentration of catecholamine in plasma (116–118). The evaluation of catecholamine in plasma has been a routine examination in clinical practice and provides convenience for clinicians to obtain knowledge of the therapeutic effect on hypertension. Nevertheless, clinicians must consider the aforementioned limitations related to clearance, sampling time, and stress when interpreting these results (65). In conclusion, the evaluation of ANS activity in patients with hypertension enhances the comprehension of the onset and development of the disease (Figure 4).

### 6.2 Treatments for hypertension induced by ANS disorders

#### 6.2.1 Modification of living habits-established therapeutic strategies

##### 6.2.1.1 Sleep

Sleep is a critical factor for the maintenance of BP, as mentioned above. Therefore, one target of therapies for BP disorders is to control abnormal sleeping states, such as OSAS. In addition to medicines such as corticosteroids (119) and tirzepatide (120), continuous positive airway pressure (CPAP) has been well established in clinical practice and widely used as an effective therapy to reduce BP in both early-onset hypertensive patients and new-onset hypertensive patients with OSAS (121, 122). As a result, CPAP decreases the overall cardiovascular risk of these patients (123). With the application of ABPM, clinicians found that CPAP could significantly reduce BP in a circadian pattern. Anabel et al. confirmed the ability of CPAP to regulate 24-h BP in patients with nocturnal hypertension and OSAS (124). A randomized cohort trial also revealed that CPAP could benefit patients with nondipping hypertension (125). Casitas et al. similarly reported that there was only a decline in nocturnal BP in patients with isolated nocturnal hypertension (INH) after treatment with CPAP, and they subsequently confirmed that CPAP could inhibit peripheral chemosensitivity by measuring the concentrations of aldosterone and diurnal catecholamines (126).

As a crucial component in the mechanisms of hypertension, catecholamines are essential indicators for evaluating the curative effects of CPAP and exploring its etiology. Even in other CVDs, such as heart failure, patients treated with CPAP were observed to have a lower concentration of catecholamine than those in the control group (127, 128). All of these studies showed that CPAP is a promising approach for treating patients with CVDs caused by ANS disorders. Certainly, as a therapy specifically aimed at patients with hypertension with OSAS, the effect of CPAP on ANS-associated hypertension still needs to be assessed in more cases along with its gradual acceptance in clinical practice (Figure 4). A key translational challenge is to identify which specific subgroups of hypertensive patients with OSAS will derive the most significant and sustained BP benefits from CPAP, beyond simply treating the sleep apnea itself. Furthermore, the long-term adherence to CPAP therapy can be a limiting factor in its overall effectiveness in real-world settings.

##### 6.2.1.2 Diet

Modification of diet, as a part of lifestyle interventions, is one of the essential approaches to treat diseases associated with nutrients. Dietary approaches to stop hypertension (DASH) were proposed under these circumstances. Saka et al. analyzed 459 volunteers without antihypertensive treatment who received 8-week DASH (rich in fruits and vegetables, low in saturated and total fat) or the control diet (low in fruits, vegetables, and average fat content), and the results indicated that patients who were assigned to DASH presented a reduction in BP (129). Some of the benefits from DASH may be due to its low levels of fat, with a less harmful effect on the cardiovascular system. In addition, the high fiber content of fruits and vegetables in DASH may also cause a transformation in the gut microbiota associated with the intestinal SNS (130). More specifically, the high fiber content can modulate the composition and metabolic activity of the gut microbiota, leading to the production of various metabolites, such as short-chain fatty acids (SCFAs). These SCFAs can interact with host cells, including enteroendocrine cells and immune cells, and may directly or indirectly influence the activity of the intestinal SNS through neural, endocrine, and immune pathways. This complex interplay, often referred to as the ‘gut-brain axis’ or ‘gut-ANS axis,’ is an emerging area of research suggesting that dietary fiber-induced changes in the gut microbiota can contribute to systemic physiological effects, including BP regulation, potentially via modulation of the ANS (131, 132). Therefore, dietary treatments for hypertension may depend on the ANS system.

As a direct cause of hypertension, salt has been the focus of many clinicians, and the benefit of a low-salt diet has been observed in clinical studies. Another DASH study to reduce sodium intake in people with high BP suggested that salt reduction in a circadian pattern had additional benefits on BP (133).

In addition to the contents of the diet, the timing of eating also influences the circadian rhythm of BP. Time-restricted feeding (TRF) is a type of intermittent fasting (IF) that restricts the fasting period to a fixed duration. It has been demonstrated that TRF can increase insulin sensitivity and improve BP in individuals with metabolic syndrome, such as those with obesity and diabetes (134–136). Hou et al. reported that BP was disordered in diabetic mice and that TRF could reverse this disorder by inhibiting sympathetic nervous activity during the day (137, 138). In addition to TRF, a sodium-restricted diet can shift the circadian rhythm of BP from a nondipper to a dipper in essential hypertension (139). These dietary interventions are supported by extensive evidence in clinical practice and are considered first-line approaches for hypertension management. These studies suggest that modifications of the diet can serve as effective therapies to treat hypertension. However, there is still not enough evidence concerning the influence of these approaches on the SNS (Figure 4).

#### 6.2.2 Pharmacologic therapies-evidence from clinical trials

Currently, pharmacologic therapies are the primary approaches used to treat hypertension. Since the evolution of antihypertensive medicines for decades, clinicians have obtained enough options to prescribe proper drugs for distinct patients. These medicines can be classified by their targets or their functions. During the medication of hypertension, drug administration at the appropriate moment is an enlightenment. Chronotherapy is a putative treatment to fight against disrupted circadian rhythms (140). They tried to optimize the timing of drug delivery to treat patients with hypertension. Hermida et al. continued to focus on this topic for further exploration and demonstrated that antihypertensive medication at night might benefit nondipping hypertensive patients (141). The results from multiple studies support this conclusion (142, 143). In 2019, a prospective trial by Hermida et al. reported that patients with hypertension who received medications before sleep had a lower cardiovascular risk (144). However, it is important to acknowledge conflicting evidence from large-scale studies. For instance, the recent Treatment in Morning vs. Evening (TIME) study, a large randomized clinical trial, found no significant difference in the risk of major cardiovascular events between patients who took their antihypertensive medication in the morning versus those who took it in the evening (145). This finding suggests that, for the general hypertensive population, the timing of antihypertensive medication may not significantly impact cardiovascular outcomes, challenging some previous observational studies and smaller trials. While the TIME study provides valuable insights, further research is needed to identify specific patient subgroups who might benefit from chronotherapy and to understand the potential reasons for the discrepancies observed across different studies. This discrepancy highlights several critical considerations. Firstly, the heterogeneity of hypertensive populations and their underlying circadian phenotypes (e.g., dippers vs. non-dippers) might influence the efficacy of chronotherapy, a factor not always adequately captured in broad clinical trials. Secondly, pharmacokinetic and pharmacodynamic properties of different antihypertensive drugs vary, and the optimal timing might be drug-specific. For example, drugs with longer half-lives might be less sensitive to timing. Thirdly, methodological differences in study design, such as patient selection criteria, duration of follow-up, and methods of blood pressure monitoring, could contribute to varied outcomes. Future research should focus on personalized chronotherapy, utilizing advanced diagnostic tools to identify individuals most likely to benefit, and exploring the optimal timing for specific drug classes based on individual circadian rhythms and genetic predispositions. Although there are no specific medical guidelines integrally developed to direct drug administration on the basis of chronotherapy, several clinical trials are in progress by cardiovascular physicians. However, *β* adrenoreceptor blockers such as metoprolol, which can reduce the activity of the SNS during treatment, have not received enough attention in the chronotherapy of hypertension. In addition, few studies have discussed the role of clock genes in the ANS, which limits our understanding of the function of the ANS in circadian rhythms during disease progression (Figure 4).

#### 6.2.3 Interventional treatment: catheter-based renal denervation-emerging interventional approaches

Kidneys are innervated by efferent nerve fibers in the SNS, and these nerve fibers are further observed in the renal cortex and the glomerular arterioles. Afferent nerves, along with renal arteries, are sensory nerves that deliver physicochemical signals such as ischemia and hypoxia to the brain and activate the whole SNS in the body (146). Subsequently, cross-talk between the renal SNS and the central SNS increases the concentration of circulating catecholamine and inversely stimulates the efferent nerves of the renal SNS, which increases BP by acting directly on the glomerular arterioles and indirectly on RASS (147, 148). Therefore, the renal SNS is crucial in the regulation of BP, and renal sympathetic denervation has been proposed as a therapy to break this loop. There are two approaches to denervating renal sympathetic nerves: surgical therapy and interventional therapy (149). Given that the method of surgery has been widely abandoned because of its multiple disadvantages, here, we discuss the method of intervention, which is usually referred to as RDN. Currently, the results of clinical studies have supported the efficacy of RDN in patients, especially those with resistant hypertension (150). Moreover, RDN not only significantly reduces 24-h BP but also significantly reduces daytime BP (151, 152), especially in patients with lower baseline BP or arterial stiffness (153, 154). At the same time, nocturnal BP was also reduced by ultrasound-based RDN (155). These clinical trials confirmed the value of RDN in regulating the circadian pattern of BP. During the application of RDN in clinical practice, a decrease in catecholamine levels was observed in the plasma of patients, which was consistent with findings in animal models (156, 157). Currently, clinical trials and fundamental experiments based on RDN are being conducted, and its benefits for hypertension elicited by ANS disorders are gradually being revealed (Figure 4). However, further studies are needed to clarify their long-term efficacy and the suitable patient population.

## 7 Potential therapeutic strategies of ANS modulation against hypertension-future and experimental therapeutic directions

### 7.1 Melatonin

Melatonin (N-acetyl-methoxy-tryptamine) is a kind of indoleamine that is a hormone secreted by the pineal glands of mammals (158). Melatonin is a messenger that delivers circadian signals in the neural circuit between the pineal glands and the SCN (159). Melatonin has been shown to be of great value for reducing BP in animal experiments. It can reduce hypoxia-mediated hypertension by preventing endothelial dysfunction and attenuating inflammation in vessels (160). In addition, melatonin was also effective in rats with stress-induced hypertension (161), and melatonin deficiency caused by pinealectomy led to continuous light-induced hypertension (162). In clinical practice, patients with nondipping hypertension or nocturnal hypertension also benefit from the administration of melatonin (163, 164). One of the main mechanisms for BP alterations during treatment is that melatonin can rebalance the ANS by increasing parasympathetic nerve activity and attenuating sympathetic nerve activity (165). In the process of ANS modulation, interference from the CNS has been proposed. Multiple regions in the brain, such as the postrema area (166), PVN (167), rostral ventrolateral medulla (RVLM) (168), and SCN (169), are involved in the modulation of the ANS. GABAergic neurons in these regions are activated by melatonin and are responsible for the inhibitory effects on the SNS (170). However, there is little evidence on the direct function of melatonin in regulating the ANS by combining with receptors such as the family of MT receptors (171) and retinoic acid-related orphan receptors (RORs) (172). Overall, melatonin provides a potential approach to treat hypertension by rebalancing the ANS, but there is still a long way to go before it can be applied (Figure 4). Despite promising preclinical and some clinical findings, the optimal dosage, formulation, and long-term safety profile of melatonin for hypertension treatment, particularly in diverse patient populations, require further rigorous investigation. The precise mechanisms by which melatonin modulates ANS activity in humans, and how these effects translate into sustained BP reduction, remain an area of active research and a key translational challenge.

### 7.2 LLTS

An imbalance of the ANS, which usually appears with the activation of the SNS and the relative weakness of the VNS, is one of the main causes of hypertension and other CVDs. Simultaneously, the anti-inflammatory properties of the VNS, which are opposite those of the SNS, have been fully recognized, accompanied by studies on the neuroimmune axis (173, 174). Thus, stimulation of the vagus nerve seems to be a straightforward way to adjust ANS disorders. Some potential therapies have been proposed, including direct vagus stimulation, baroreceptor stimulation, and LLTS (175). However, the disadvantages of invasive operations and device implantation restrict the development of vagus stimulation. Techniques that target the auricular branch of the vagus nerve (ABVN) represent a promising approach (176). LLTS applied to ABVN has been reported to improve and reverse CVDs in different phases. Recently, models of myocardial ischemia (MI) have been developed. Research has verified the efficiency of chronic LLTS in attenuating left ventricle (LV) remodeling, reducing ventricular arrhythmia inducibility and inhibiting left stellate ganglia (LSG) neural activity (177–179). In addition, acute LLTS also performed well in a model of atrial fibrillation (AF) by reversing rapid atrial pacing-induced atrial remodeling and inhibiting AF inducibility (180, 181). However, few studies have reported the value of LLTS in the treatment of hypertension, although Zhou et al. reported a smaller increase in both SBP and DBP in HFpEF rats treated with LLTS than in control rats (182). Remarkably, the shortage of evidence for LLTS in hypertension may be attributed to the fact that most of the studies above selected the parameters of stimulation below the heart rate slowing threshold. Although this action ensured safety from basic to clinical transformation, it also prevented us from knowing the effects of LLTS on BP with subthreshold stimulation. This represents a significant methodological limitation, as the optimal stimulation parameters for achieving a clinically meaningful antihypertensive effect without adverse events are yet to be fully elucidated. The translational gap here lies in optimizing LLTS protocols for hypertension specifically, moving beyond its established benefits in other cardiovascular conditions. In summary, an increasing number of studies have shown the possibility of LLTS to cure hypertension (Figure 4).

### 7.3 Optogenetics

Given the importance of the SNS in the cardiovascular system, experts in the field have focused on therapies for CVD by precisely inhibiting the activity of the SNS. The LSG is a type of cervical sympathetic ganglion combined with the inferior cervical ganglion and the first pair of thoracic sympathetic ganglia. The nerve fibers projected from the LSG innervate a wide range of nerves, including the eyelid, pupil, capillary, and even the heart, via the formation of the cardiac plexus. Studies have shown that LSG ablation and suppression protect against cardiac arrhythmia, such as ventricular fibrillation after AMI, and improve the outcomes of patients with malignant CVD (183, 184). However, there is still no therapy based on LSG modulation that integrates theories of circadian rhythms.

The development of optogenetics may provide a new method for the nonpharmacological treatment of hypertension. In a canine AMI model, an adeno-associated virus (AAV) was used as a carrier to deliver the inhibitory photosensitive protein ArchT to LSG neurons, and researchers reported that ischemia-induced ventricular arrhythmia was significantly suppressed by the simultaneous inhibition of LSG activity and LSG function during transient light-emitting diode illumination and the evaluation of BP changes (185). These results demonstrated that the application of optogenetic technology to decrease the risk of malignant CVDs is remarkable. This means that precise modulation of the ANS is available according to the physical and psychological conditions of patients with CVD. The characteristics of intervention at the appropriate time point are quite consistent with the concept of chronotherapy for hypertension. Given the patterns of hypertension, clinical trials are needed to screen appropriate populations integrated with human genomics and develop individualized programs to provide suitable light intensity at the proper time. Although there is still room for improvement, we look forward to optogenetics becoming a promising therapy for modulating the ANS to protect against hypertension (Figure 4).

## 8 Conclusion and future perspectives

Hypertension is the most common cardiovascular disease and is also the leading contributor to multiple fatal illnesses. In recent decades, scientists have explored the potential mechanisms of BP modulation and focused on curing hypertension. Consequently, the ANS has been proposed as an important factor that regulates BP in a circadian pattern. Using techniques of histology, scientists have deconstructed circadian clocks and discovered that the ANS is the bridge between the central clock and peripheral clocks. Although the ANS is a considerable factor involved in the occurrence of hypertension, the molecular mechanisms underlying ANS tone changes during the day remain to be clarified. Fortunately, with the development of molecular biology, genes that modulate fluctuations in ANS activity will be identified, and whether these genes contribute to BP disorders will be ascertained. It is predictable that the above genes will act as effective targets for treating hypertension in clinical practice and that therapies targeting these genes will surely provide hope for patients with hypertension.

## References

[1] Ding JM Chen D Weber ET Faiman LE Rea MA Gillette MU. Resetting the biological clock: mediation of nocturnal circadian shifts by glutamate and NO. Science. (1994) 266:1713–7. 7527589 10.1126/science.7527589

[2] Kalsbeek A Palm IF La Fleur SE Scheer FAJL Perreau-Lenz S Ruiter M . SCN outputs and the hypothalamic balance of life. J Biol Rhythm. (2006) 21:458–69. doi: 10.1177/0748730406293854, 17107936

[3] Moore RY. Circadian rhythms: basic neurobiology and clinical applications. Annu Rev Med. (1997) 48:253–66. 9046960 10.1146/annurev.med.48.1.253

[4] Mohawk JA Green CB Takahashi JS. Central and peripheral circadian clocks in mammals. Annu Rev Neurosci. (2012) 35:445–62. doi: 10.1146/annurev-neuro-060909-153128, 22483041 PMC3710582

[5] Bell-Pedersen D Cassone VM Earnest DJ Golden SS Hardin PE Thomas TL. Circadian rhythms from multiple oscillators: lessons from diverse organisms. Nat Rev Genet. (2005) 6:544–56. doi: 10.1038/nrg1633, 15951747 PMC2735866

[6] Kriegsfeld LJ Silver R. The regulation of neuroendocrine function: timing is everything. Horm Behav. (2006) 49:557–74. doi: 10.1016/j.yhbeh.2005.12.011, 16497305 PMC3275441

[7] Gamble KL Berry R Frank SJ Young ME. Circadian clock control of endocrine factors. Nat Rev Endocrinol. (2014) 10:466–75. doi: 10.1038/nrendo.2014.78, 24863387 PMC4304769

[8] Schrader LA Ronnekleiv-Kelly SM Hogenesch JB Bradfield CA Malecki KMC. Circadian disruption, clock genes, and metabolic health. J Clin Invest. (2024) 134:e170998. doi: 10.1172/JCI170998, 39007272 PMC11245155

[9] Yu Z Liu Z Jiao L Zhang S Nie L Wang Y . Bmal1 knockdown in the left stellate ganglion inhibits neural activity and prevents ventricular arrhythmias after myocardial ischemia. Front Cardiovasc Med. (2022) 9:937608. doi: 10.3389/fcvm.2022.937608, 36247430 PMC9556266

[10] Kim P Oster H Lehnert H Schmid SM Salamat N Barclay JL. Coupling the circadian clock to homeostasis: the role of period in timing physiology. Endocr Rev. (2019) 40:66–95. doi: 10.1210/er.2018-00049, 30169559

[11] Degaute JP Vandeborne P Linkowski P Vancauter E. Quantitative-analysis of the 24-hour blood-pressure and heart-rate patterns in Young men. Hypertension. (1991) 18:199–210. 1885228 10.1161/01.hyp.18.2.199

[12] Bianchi S Bigazzi X Baldari G Sgherri G Campese VM. Diurnal-variations of blood-pressure and microalbuminuria in essential-hypertension. Am J Hypertens. (1994) 7:23–9. 8136107 10.1093/ajh/7.1.23

[13] Lin Y-H Chen Y-C Chen J-Y. Disrupted circadian rhythm as a mediator of autonomic dysregulation and overactive bladder in men with benign prostatic hyperplasia. Eur Urol Focus. (2025) 12:144–6. doi: 10.1016/j.euf.2025.05.001, 40368721

[14] Cuspidi C Sala C Tadic M Gherbesi E De Giorgi A Grassi G . Clinical and prognostic significance of a reverse dipping pattern on ambulatory monitoring: an updated review. J Clin Hypertens. (2017) 19:713–21. doi: 10.1111/jch.13023, 28692165 PMC8031119

[15] Salles GF Reboldi G Fagard RH Cardoso CRL Pierdomenico SD Verdecchia P . Prognostic effect of the nocturnal blood pressure fall in hypertensive patients the ambulatory blood pressure collaboration in patients with hypertension (ABC-H) Meta-analysis. Hypertension. (2016) 67:693–700. doi: 10.1161/Hypertensionaha.115.06981, 26902495

[16] Habas E Akbar RA Alfitori G Farfar KL Habas E Errayes N. Effects of nondipping blood pressure changes: a nephrologist Prospect. Cureus. (2023) 15:e42681. doi: 10.7759/cureus.42681, 37649932 PMC10464654

[17] Woon PY Kaisaki PJ Bragança J Bihoreau MT Levy JC Farrall M. Aryl hydrocarbon receptor nuclear translocator-like (BMAL1) is associated with susceptibility to hypertension and type 2 diabetes. P Natl Acad Sci USA. (2007) 104:14412–7. doi: 10.1073/pnas.0703247104, 17728404 PMC1958818

[18] Curtis AM Cheng Y Kapoor S Reilly D Price TS FitzGerald GA. Circadian variation of blood pressure and the vascular response to asynchronous stress. P Natl Acad Sci USA. (2007) 104:3450–5. doi: 10.1073/pnas.0611680104, 17360665 PMC1802007

[19] Xie ZW Su W Liu S Zhao GG Esser K Schroder EA . Smooth-muscle BMAL1 participates in blood pressure circadian rhythm regulation. J Clin Invest. (2015) 125:324–36. doi: 10.1172/Jci76881, 25485682 PMC4382248

[20] Marques FZ Campain AE Tomaszewski M Zukowska-Szczechowska E Yang YHJ Charchar FJ. Gene expression profiling reveals renin mRNA overexpression in human hypertensive kidneys and a role for MicroRNAs. Hypertension. (2011) 58:1093–8. doi: 10.1161/Hypertensionaha.111.180729, 22042811

[21] Stow LR Richards J Cheng KY Lynch IJ Jeffers LA Greenlee MM. The circadian protein period 1 contributes to blood pressure control and coordinately regulates renal sodium transport genes. Hypertension. (2012) 59:1151–6. doi: 10.1161/Hypertensionaha.112.190892, 22526258 PMC3366275

[22] Sei H Oishi K Chikahisa S Kitaoka K Takeda E Ishida N. Diurnal amplitudes of arterial pressure and heart rate are dampened in mutant mice and adrenalectomized mice. Endocrinology. (2008) 149:3576–80. doi: 10.1210/en.2007-1714, 18403480

[23] Doi M Takahashi Y Komatsu R Yamazaki F Yamada H Haraguchi S. Salt-sensitive hypertension in circadian clock-deficient -null mice involves dysregulated adrenal Hsd3b6. Nat Med. (2010) 16:67–74. doi: 10.1038/nm.2061, 20023637

[24] Krasemann T Strompen C Blumenberg J Gehrmann J Burkhardtsmaier G Vogt J. Changes of the corrected QT interval in healthy boys and girls over day and night. Eur Heart J. (2009) 30:202–8. doi: 10.1093/eurheartj/ehn452, 18832384

[25] Vukolic A Antic V Van Vliet BN Yang ZH Albrecht U Montani JP. Role of mutation of the circadian clock gene in cardiovascular circadian rhythms. Am J Physiol-Reg I. (2010) 298:R627–34. doi: 10.1152/ajpregu.00404.2009, 20053965

[26] Morris CJ Purvis TE Hu K Scheer FAJL. Circadian misalignment increases cardiovascular disease risk factors in humans. P Natl Acad Sci USA. (2016) 113:E1402–11. doi: 10.1073/pnas.1516953113, 26858430 PMC4790999

[27] Morris CJ Purvis TE Mistretta J Hu K Scheer FAJL. Circadian misalignment increases C-reactive protein and blood pressure in chronic shift workers. J Biol Rhythm. (2017) 32:154–64. doi: 10.1177/0748730417697537, 28347188 PMC5858578

[28] Sutovska HM Obermajer V Zeman M Molcan L. Artificial light at night affects the daily profile of pulse pressure and protein expression in the thoracic aorta of rats. Hypertens Res. (2024) 47:1897–907. doi: 10.1038/s41440-024-01685-9, 38664509 PMC11224016

[29] Molcan L Sutovska H Okuliarova M Senko T Krskova L Zeman M. Dim light at night attenuates circadian rhythms in the cardiovascular system and suppresses melatonin in rats. Life Sci. (2019) 231:116568. doi: 10.1016/j.lfs.2019.116568, 31202842

[30] Rumanova VS Okuliarova M Molcan L Sutovska H Zeman M. Consequences of low-intensity light at night on cardiovascular and metabolic parameters in spontaneously hypertensive rats. Can J Physiol Pharmacol. (2019) 97:863–71. doi: 10.1139/cjpp-2019-0043, 31251886

[31] Prabhat A Sami D Ehlman A Stumpf I Seward T Su W . Dim light at night unmasks sex-specific differences in circadian and autonomic regulation of cardiovascular physiology. Commun Biol. (2024) 7:1191. doi: 10.1038/s42003-024-06861-8 39333678 PMC11437115

[32] Franken P Dijk DJ. Sleep and circadian rhythmicity as entangled processes serving homeostasis. Nat Rev Neurosci. (2024) 25:43–59. doi: 10.1038/s41583-023-00764-z, 38040815

[33] Li Y Staessen JA Lu L Li LH Wang GL Wang JG. Is isolated nocturnal hypertension a novel clinical entity? Findings from a Chinese population study. Hypertension. (2007) 50:333–9. doi: 10.1161/Hypertensionaha.107.087767, 17576859

[34] Boggia J Li Y Thijs L Hansen TW Kikuya M Björklund-Bodegård K . Prognostic accuracy of day versus night ambulatory blood pressure: a cohort study. Lancet. (2007) 370:1219–29. doi: 10.1016/S0140-6736(07)61538-4 17920917

[35] Li Y Wang JG. Isolated nocturnal hypertension a disease masked in the dark. Hypertension. (2013) 61:278–83. doi: 10.1161/Hypertensionaha.111.00217, 23248146

[36] Hou HF Zhao YG Yu WQ Dong HL Xue XT Ding J . Association of obstructive sleep apnea with hypertension: a systematic review and meta-analysis. J Glob Health. (2018) 8:010405. doi: 10.7189/jogh.08.010405 29497502 PMC5825975

[37] Kapur VK Auckley DH Chowdhuri S Kuhlmann DC Mehra R Ramar K. Clinical practice guideline for diagnostic testing for adult obstructive sleep apnea: an American Academy of sleep medicine clinical practice guideline. J Clin Sleep Med. (2017) 13:479–504. doi: 10.5664/jcsm.6506, 28162150 PMC5337595

[38] Pedrosa RP Drager LF Gonzaga CC Sousa MG de Paula LKG Amaro ACS. Obstructive sleep apnea the Most common secondary cause of hypertension associated with resistant hypertension. Hypertension. (2011) 58:811–7. doi: 10.1161/Hypertensionaha.111.179788, 21968750

[39] Martínez-García MA Navarro-Soriano C Torres G Barbé F Caballero-Eraso C Lloberes P. Beyond resistant hypertension: relationship between refractory hypertension and obstructive sleep apnea. Hypertension. (2018) 72:618–24. doi: 10.1161/Hypertensionaha.118.11170, 30354751

[40] Amin RS Carroll JL Jeffries JL Grone C Bean JA Chini B . Twenty-four-hour ambulatory blood pressure in children with sleep-disordered breathing. Am J Respir Crit Care Med. (2004) 169:950–6. doi: 10.1164/rccm.200309-1305OC 14764433

[41] Poggiogalle E Jamshed H Peterson CM. Circadian regulation of glucose, lipid, and energy metabolism in humans. Metabolism. (2018) 84:11–27. doi: 10.1016/j.metabol.2017.11.017, 29195759 PMC5995632

[42] Gooley JJ. Circadian regulation of lipid metabolism. P Nutr Soc. (2016) 75:440–50. doi: 10.1017/S0029665116000288, 27225642

[43] MacDonald A Singh RH Rocha JC van Spronsen FJ. Optimising amino acid absorption: essential to improve nitrogen balance and metabolic control in phenylketonuria. Nutr Res Rev. (2019) 32:70–8. doi: 10.1017/S0954422418000173, 30284526 PMC6536823

[44] Magkos F Hjorth MF Astrup A. Diet and exercise in the prevention and treatment of type 2 diabetes mellitus. Nat Rev Endocrinol. (2020) 16:545–55. doi: 10.1038/s41574-020-0381-5, 32690918

[45] Pagidipati NJ Taub PR Ostfeld RJ Kirkpatrick CF. Dietary patterns to promote cardiometabolic health. Nat Rev Cardiol. (2024) 22:38–46. doi: 10.1038/s41569-024-01061-7, 39020052

[46] Saxton SN Clark B Withers SB Eringa EC Heagerty AM. Mechanistic links between obesity, diabetes, and blood pressure: role of perivascular adipose tissue. Physiol Rev. (2019) 99:1701–63. doi: 10.1152/physrev.00034.2018, 31339053

[47] Erdos B Broxson CS Cudykier I Basgut B Whidden M Landa T. Effect of high-fat diet feeding on hypothalamic redox signaling and central blood pressure regulation. Hypertens Res. (2009) 32:983–8. doi: 10.1038/hr.2009.129, 19713964

[48] Carroll JF Thaden JJ Wright AM Strange T. Loss of diurnal rhythms of blood pressure and heart rate caused by high-fat feeding. Am J Hypertens. (2005) 18:1320–6. doi: 10.1016/j.amjhyper.2005.04.018, 16202855

[49] Siri-Tarino PW Sun Q Hu FB Krauss RM. Meta-analysis of prospective cohort studies evaluating the association of saturated fat with cardiovascular disease. Am J Clin Nutr. (2010) 91:535–46. doi: 10.3945/ajcn.2009.27725, 20071648 PMC2824152

[50] Haruhara K Tsuboi N Koike K Fukui A Miyazaki Y Kawamura T. Renal histopathological findings in relation to ambulatory blood pressure in chronic kidney disease patients. Hypertens Res. (2015) 38:116–22. doi: 10.1038/hr.2014.140, 25231252

[51] Sufiun A Rahman A Rafiq K Fujisawa Y Nakano D Kobara H. Association of a Disrupted Dipping Pattern of blood pressure with progression of renal injury during the development of salt-dependent hypertension in rats. Int J Mol Sci. (2020) 21:2248. doi: 10.3390/ijms21062248 32213948 PMC7139748

[52] Gupta DK Lewis CE Varady KA Su YR Madhur MS Lackland DT. Effect of dietary sodium on blood pressure: a crossover trial. JAMA. (2023) 330:2258–66. doi: 10.1001/jama.2023.23651, 37950918 PMC10640704

[53] Sztajzel J. Heart rate variability: a noninvasive electrocardiographic method to measure the autonomic nervous system. Swiss Med Wkly. (2004) 134:514–22. doi: 10.4414/smw.2004.10321 15517504

[54] Han S Kobayashi K Joung B Piccirillo G Maruyama M Vinters HV. Electroanatomic remodeling of the left stellate ganglion after myocardial infarction. J Am Coll Cardiol. (2012) 59:954–61. doi: 10.1016/j.jacc.2011.11.030, 22381432 PMC3975658

[55] Ogawa M Zhou SM Tan AY Song J Gholmieh G Fishbein MC . Left stellate ganglion and vagal nerve activity and cardiac arrhythmias in ambulatory dogs with pacing-induced congestive heart failure. J Am Coll Cardiol. (2007) 50:335–43. doi: 10.1016/j.jacc.2007.03.045, 17659201

[56] Aston-Jones G Chen S Zhu Y Oshinsky ML. A neural circuit for circadian regulation of arousal. Nat Neurosci. (2001) 4:732–8. doi: 10.1038/89522 11426230

[57] Guyenet PG. The sympathetic control of blood pressure. Nat Rev Neurosci. (2006) 7:335–46. doi: 10.1038/nrn1902, 16760914

[58] Zhang DY Anderson AS. The sympathetic nervous system and heart failure. Cardiol Clin. (2014) 32:33. doi: 10.1016/j.ccl.2013.09.010, 24286577 PMC5873965

[59] DiBona GF. Neural control of the kidney - past, present, and future. Hypertension. (2003) 41:621–4. doi: 10.1161/01.Hyp.0000047205.52509.8a, 12623969

[60] Hart EC Charkoudian N. Sympathetic neural mechanisms in human blood pressure regulation. Curr Hypertens Rep. (2011) 13:237–43. doi: 10.1007/s11906-011-0191-1, 21293977

[61] Grassi G Ram VS. Evidence for a critical role of the sympathetic nervous system in hypertension. J Am Soc Hypertens. (2016) 10:457–66. doi: 10.1016/j.jash.2016.02.015, 27052349

[62] Benarroch EE. Physiology and pathophysiology of the autonomic nervous system. Continuum (Minneap Minn). (2020) 26:12–24. doi: 10.1212/con.0000000000000817, 31996619

[63] Esler M. The sympathetic nervous system through the ages: from Thomas Willis to resistant hypertension. Exp Physiol. (2011) 96:611–22. doi: 10.1113/expphysiol.2011.052332, 21551268

[64] Grassi G Mark A Esler M. The sympathetic nervous system alterations in human hypertension. Circ Res. (2015) 116:976–90. doi: 10.1161/Circresaha.116.303604, 25767284 PMC4367954

[65] Chen Y Xiao H Zhou X Huang X Li Y Xiao H. Accuracy of plasma free METANEPHRINES in the diagnosis of PHEOCHROMOCYTOMA and PARAGANGLIOMA: a systematic review and META-analysis. Endocr Pract. (2017) 23:1169–77. doi: 10.4158/EP171877.OR, 28704098

[66] Oparil S Acelajado MC Bakris GL Berlowitz DR Cifkova R Dominiczak AF . Hypertension. Nat Rev Dis Primers. (2018) 4:18014. doi: 10.1038/nrdp.2018.14, 29565029 PMC6477925

[67] Pamphlett R Jew SK Doble PA Bishop DP. Mercury in the human adrenal medulla could contribute to increased plasma noradrenaline in aging. Sci Rep-Uk. (2021) 11:2961. doi: 10.1038/s41598-021-82483-y PMC7858609 33536525

[68] Fujita T. Mechanism of salt-sensitive hypertension: focus on adrenal and sympathetic nervous systems. J Am Soc Nephrol. (2014) 25:1148–55. doi: 10.1681/Asn.2013121258, 24578129 PMC4033384

[69] Duan W Ye P Leng YQ Liu DH Sun JC Tan X. Oxidative stress in the RVLM mediates sympathetic hyperactivity induced by circadian disruption. Neurosci Lett. (2022) 791:136917. doi: 10.1016/j.neulet.2022.136917, 36252850

[70] Briaud SA Zhang BL Sannajust F. Continuous light exposure and sympathectomy suppress circadian rhythm of blood pressure in rats. J Cardiovasc Pharm T. (2004) 9:97–105. doi: 10.1177/107424840400900205, 15309246

[71] Souza BB Monteze NM de Oliveira FLP de Oliveira JM de Freitas SN Neto RMD . Lifetime shift work exposure: association with anthropometry, body composition, blood pressure, glucose and heart rate variability. Occup Environ Med. (2015) 72:208–15. doi: 10.1136/oemed-2014-102429, 25540411

[72] Rauchenzauner M Ernst F Hintringer F Ulmer H Ebenbichler CF Kasseroler MT. Arrhythmias and increased neuro-endocrine stress response during physicians' night shifts: a randomized cross-over trial. Eur Heart J. (2009) 30:2606–13. doi: 10.1093/eurheartj/ehp268, 19602503

[73] Steptoe A Kivimäki M. Stress and cardiovascular disease: an update on current knowledge. Annu Rev Public Health. (2013) 34:337–54. doi: 10.1146/annurev-publhealth-031912-114452, 23297662

[74] Chau NP Bauduceau B Chanudet X Larroque P Gautier D. Ambulatory blood-pressure in diabetic subjects. Am J Hypertens. (1994) 7:487–91. 7917144

[75] Smolensky MH Hermida RC Portaluppi F Haus E. Twenty-four-hour pattern of angina pectoris, acute myocardial infarction and sudden cardiac death: role of blood pressure, heart rate and rate-pressure product circadian rhythms. Biol Rhythm Res. (2007) 38:205–16. doi: 10.1080/09291010600906166

[76] Portaluppi F Cortelli P Avoni P Vergnani L Contin M Maltoni P. Diurnal blood-pressure variation and hormonal correlates in fatal familial insomnia. Hypertension. (1994) 23:569–76. 8175163 10.1161/01.hyp.23.5.569

[77] Somers VK Dyken ME Clary MP Abboud FM. Sympathetic neural mechanisms in obstructive sleep-apnea. J Clin Invest. (1995) 96:1897–904. 7560081 10.1172/JCI118235 PMC185826

[78] Lesske J Fletcher EC Bao G Unger T. Hypertension caused by chronic intermittent hypoxia - influence of chemoreceptors and sympathetic nervous system. J Hypertens. (1997) 15:1593–603. 9488210 10.1097/00004872-199715120-00060

[79] Fang ZW Carlson SH Peng N Wyss JM. Circadian rhythm of plasma sodium is disrupted in spontaneously hypertensive rats fed a high-NaCl diet. Am J Physiol-Reg I. (2000) 278:R1490–5. 10.1152/ajpregu.2000.278.6.R1490 10848515

[80] Zhang M Qin DN Suo YP Su Q Li HB Miao YW. Endogenous hydrogen peroxide in the hypothalamic paraventricular nucleus regulates neurohormonal excitation in high salt-induced hypertension. Toxicol Lett. (2015) 235:206–15. doi: 10.1016/j.toxlet.2015.04.008, 25891026

[81] Osanai T Okuguchi T Kamada T Fujiwara N Kosugi T Saitoh G. Salt-induced exacerbation of morning surge in blood pressure in patients with essential hypertension. J Hum Hypertens. (2000) 14:57–64. 10673733 10.1038/sj.jhh.1000945

[82] Carlson SH Roysomutti S Peng N Wyss JM. The role of the central nervous system in NaCl-sensitive hypertension in spontaneously hypertensive rats. Am J Hypertens. (2001) 14:S155–62. doi: 10.1016/S0895-7061(01)02083-0 11411751

[83] Farquhar WB Edwards DG Jurkovitz CT Weintraub WS. Dietary sodium and health more than just blood pressure. J Am Coll Cardiol. (2015) 65:1042–50. doi: 10.1016/j.jacc.2014.12.039, 25766952 PMC5098396

[84] Stocker SD Madden CJ Sved AF. Excess dietary salt intake alters the excitability of central sympathetic networks. Physiol Behav. (2010) 100:519–24. doi: 10.1016/j.physbeh.2010.04.024, 20434471 PMC3024145

[85] Petrie JR Guzik TJ Touyz RM. Diabetes, hypertension, and cardiovascular disease: clinical insights and vascular mechanisms. Can J Cardiol. (2018) 34:575–84. doi: 10.1016/j.cjca.2017.12.005, 29459239 PMC5953551

[86] Farah V Elased KM Chen YF Key MP Cunha TS Irigoyen MC . Nocturnal hypertension in mice consuming a high fructose diet. Auton Neurosci-Basic. (2006) 130:41–50. doi: 10.1016/j.autneu.2006.05.006, 16843071

[87] Bray GA. Energy and fructose from beverages sweetened with sugar or high-fructose corn syrup pose a health risk for some people. Adv Nutr. (2013) 4:220–5. doi: 10.3945/an.112.002816, 23493538 PMC3649102

[88] Fukuda M Urushihara M Wakamatsu T Oikawa T Kobori H. Proximal tubular angiotensinogen in renal biopsy suggests nondipper BP rhythm accompanied by enhanced tubular sodium reabsorption. J Hypertens. (2012) 30:1453–9. doi: 10.1097/HJH.0b013e328353e807, 22573118 PMC3378825

[89] Gordon RD Wolfe LK Island DP Liddle GW. A diurnal rhythm in plasma renin activity in man. J Clin Invest. (1966) 45:1587–92. 4288714 10.1172/JCI105464 PMC292839

[90] Kala R Fyhrquist F Eisalo A. Diurnal variation of plasma angiotensin II in man. Scand J Clin Lab Invest. (1973) 31:363–5. 4357530 10.3109/00365517309084318

[91] Tuo P Zhao RS Li N Yan S Yang GG Wang CM . Lycorine inhibits Ang II-induced heart remodeling and inflammation by suppressing the PI3K-AKT/NFκ B pathway. Phytomedicine. (2024) 128:155464. doi: 10.1016/j.phymed.2024.155464, 38484625

[92] Isobe S Ohashi N Fujikura T Tsuji T Sakao Y Yasuda H. Disturbed circadian rhythm of the intrarenal renin-angiotensin system: relevant to nocturnal hypertension and renal damage. Clin Exp Nephrol. (2015) 19:231–9. doi: 10.1007/s10157-014-0973-2, 24728489

[93] Tain YL Lin YJ Sheen JM Yu HR Tiao MM Chen CC . High fat diets sex-specifically affect the renal transcriptome and program obesity, kidney injury, and hypertension in the offspring. Nutrients. (2017) 9:357. doi: 10.3390/nu9040357 28368364 PMC5409696

[94] Fedorova OV Shilova VY Zernetkina V Juhasz O Wei W Lakatta EG. Silencing of PKG1 gene mimics effect of aging and sensitizes rat vascular smooth muscle cells to Cardiotonic steroids: impact on fibrosis and salt sensitivity. J Am Heart Assoc. (2023) 12:e028768. doi: 10.1161/jaha.122.028768, 37301747 PMC10356040

[95] Sothern RB Vesely DL Kanabrocki EL Hermida RC Bremner FW Third JLHC. Temporal (circadian) and functional-relationship between atrial natriuretic peptides and blood-pressure. Chronobiol Int. (1995) 12:106–20. 8653797 10.3109/07420529509064506

[96] Volpe M Battistoni A Rubattu S. Natriuretic peptides in heart failure: current achievements and future perspectives. Int J Cardiol. (2018) 281:186–9. doi: 10.1016/j.ijcard.2018.04.045, 30545616

[97] Brunner-La Rocca HP Kaye DM Woods RL Hastings J Esler MD. Effects of intravenous brain natriuretic peptide on regional sympathetic activity in patients with chronic heart failure as compared with healthy control subjects. J Am Coll Cardiol. (2001) 37:1221–7. doi: 10.1016/s0735-1097(01)01172-x 11300426

[98] Parcha V Patel N Gutierrez OM Li P Gamble KL Musunuru K. Chronobiology of natriuretic peptides and blood pressure in lean and obese individuals. J Am Coll Cardiol. (2021) 77:2291–303. doi: 10.1016/j.jacc.2021.03.291, 33958126 PMC8138944

[99] Tamura S Iwata S Ito A Ishikawa S Mizutani K Izumiya Y. Greater nocturnal blood pressure is associated with natriuretic peptide level in aortic stenosis with preserved ejection fraction. Circ J. (2019) 83:447–51. doi: 10.1253/circj.CJ-18-0818, 30464111

[100] Guzik TJ Touyz RM. Oxidative stress, inflammation, and vascular aging in hypertension. Hypertension (Dallas, Tex: 1979). (2017) 70:660–7. doi: 10.1161/HYPERTENSIONAHA.117.07802 28784646

[101] Mazzali M Hughes J Kim YG Jefferson JA Kang DH Gordon KL . Elevated uric acid increases blood pressure in the rat by a novel crystal-independent mechanism. Hypertension (Dallas, Tex: 1979). (2001) 38:1101–6. doi: 10.1016/s0735-1097(01)01172-x 11711505

[102] Saederup N Chan L Lira SA Charo IF. Fractalkine deficiency markedly reduces macrophage accumulation and atherosclerotic lesion formation in CCR2−/− mice: evidence for independent chemokine functions in atherogenesis. Circulation. (2008) 117:1642–8. doi: 10.1161/circulationaha.107.743872, 18165355 PMC3589525

[103] Carnevale D Perrotta M Pallante F Fardella V Iacobucci R Fardella S . A cholinergic-sympathetic pathway primes immunity in hypertension and mediates brain-to-spleen communication. Nat Commun. (2016) 7:13035. doi: 10.1038/ncomms13035, 27676657 PMC5052663

[104] Wei B Cheng G Bi Q Lu C Sun Q Li L. Microglia in the hypothalamic paraventricular nucleus sense hemodynamic disturbance and promote sympathetic excitation in hypertension. Immunity. (2024) 57:2030–2042.e8. doi: 10.1016/j.immuni.2024.07.011, 39116878

[105] Boos CJ Toon LT Almahdi H. The relationship between ambulatory arterial stiffness, inflammation, blood pressure dipping and cardiovascular outcomes. BMC Cardiovasc Disord. (2021) 21:139. doi: 10.1186/s12872-021-01946-2, 33726683 PMC7968202

[106] Kanki M Nath AP Xiang R Yiallourou S Fuller PJ Cole TJ. Poor sleep and shift work associate with increased blood pressure and inflammation in UK biobank participants. Nat Commun. (2023) 14:7096. doi: 10.1038/s41467-023-42758-6, 37925459 PMC10625529

[107] Benjamin EJ Blaha MJ Chiuve SE Cushman M Das SR Deo R . Heart disease and stroke Statistics-2017 update: a report from the American Heart Association. Circulation. (2017) 135:e146–603. doi: 10.1161/cir.0000000000000485, 28122885 PMC5408160

[108] Dedhia RC Bliwise DL Quyyumi AA Thaler ER Boon MS Huntley CT. Hypoglossal nerve stimulation and cardiovascular outcomes for patients with obstructive sleep apnea: a randomized clinical trial. JAMA Otolaryngol Head Neck Surg. (2024) 150:39–48. doi: 10.1001/jamaoto.2023.3756, 38032624 PMC10690581

[109] Kario K Hoshide S Mizuno H Kabutoya T Nishizawa M Yoshida T. Nighttime blood pressure phenotype and cardiovascular prognosis: practitioner-based Nationwide JAMP study. Circulation. (2020) 142:1810–20. doi: 10.1161/circulationaha.120.049730, 33131317 PMC7643792

[110] Wong ND Dede J Chow VH Wong KS Franklin SS. Global cardiovascular risk associated with hypertension and extent of treatment and control according to risk group. Am J Hypertens. (2012) 25:561–7. doi: 10.1038/ajh.2012.2, 22318511

[111] Oh J Lee CJ Kim IC Lee SH Kang SM Choi D . Association of Morning Hypertension Subtype with Vascular Target Organ Damage and Central Hemodynamics. J Am Heart Assoc. (2017) 6:e005424. doi: 10.1161/jaha.116.005424, 28196818 PMC5523792

[112] Huang QF Yang WY Asayama K Zhang ZY Thijs L Li Y. Ambulatory blood pressure monitoring to diagnose and manage hypertension. Hypertension. (2021) 77:254–64. doi: 10.1161/hypertensionaha.120.14591, 33390042 PMC7803442

[113] Grassi G Seravalle G Quarti-Trevano F Dell'Oro R Bombelli M Cuspidi C. Adrenergic, metabolic, and reflex abnormalities in reverse and extreme dipper hypertensives. Hypertension. (2008) 52:925–31. doi: 10.1161/hypertensionaha.108.116368, 18779438

[114] de Barros S da Silva GV de Gusmão JL de Araújo TG de Souza DR Cardoso CG Jr. Effects of long term device-guided slow breathing on sympathetic nervous activity in hypertensive patients: a randomized open-label clinical trial. Blood Press. (2017) 26:359–65. doi: 10.1080/08037051.2017.1357109, 28724309

[115] Okada Y Galbreath MM Shibata S Jarvis SS Bivens TB Vongpatanasin W. Morning blood pressure surge is associated with arterial stiffness and sympathetic baroreflex sensitivity in hypertensive seniors. Am J Physiol Heart Circ Physiol. (2013) 305:H793–802. doi: 10.1152/ajpheart.00254.2013, 23832695 PMC3761347

[116] Siddiqui M Judd EK Jaeger BC Bhatt H Dudenbostel T Zhang B. Out-of-clinic sympathetic activity is increased in patients with masked uncontrolled hypertension. Hypertension. (2019) 73:132–41. doi: 10.1161/hypertensionaha.118.11818, 30571547 PMC6309788

[117] Gilardini L Parati G Sartorio A Mazzilli G Pontiggia B Invitti C. Sympathoadrenergic and metabolic factors are involved in ambulatory blood pressure rise in childhood obesity. J Hum Hypertens. (2008) 22:75–82. doi: 10.1038/sj.jhh.1002288, 17882228

[118] García-Río F Pino JM Alonso A Arias MA Martínez I Alvaro D. White coat hypertension in patients with obstructive sleep apnea-hypopnea syndrome. Chest. (2004) 125:817–22. doi: 10.1378/chest.125.3.817, 15006937

[119] Tapia IE Shults J Cielo CM Kelly AB Elden LM Spergel JM. A trial of intranasal corticosteroids to treat childhood OSA syndrome. Chest. (2022) 162:899–919. doi: 10.1016/j.chest.2022.06.026, 35779610 PMC9633812

[120] Malhotra A Grunstein RR Fietze I Weaver TE Redline S Azarbarzin A. Tirzepatide for the treatment of obstructive sleep apnea and obesity. N Engl J Med. (2024) 391:1193–205. doi: 10.1056/NEJMoa2404881, 38912654 PMC11598664

[121] Marin JM Carrizo SJ Vicente E Agusti AG. Long-term cardiovascular outcomes in men with obstructive sleep apnoea-hypopnoea with or without treatment with continuous positive airway pressure: an observational study. Lancet. (2005) 365:1046–53. doi: 10.1016/s0140-6736(05)71141-7 15781100

[122] Marin JM Agusti A Villar I Forner M Nieto D Carrizo SJ. Association between treated and untreated obstructive sleep apnea and risk of hypertension. JAMA. (2012) 307:2169–76. doi: 10.1001/jama.2012.3418, 22618924 PMC4657563

[123] Barbé F Durán-Cantolla J Capote F de la Peña M Chiner E Masa JF. Long-term effect of continuous positive airway pressure in hypertensive patients with sleep apnea. Am J Respir Crit Care Med. (2010) 181:718–26. doi: 10.1164/rccm.200901-0050OC 20007932

[124] Castro-Grattoni AL Torres G Martínez-Alonso M Barbé F Turino C Sánchez-de-la-Torre A. Blood pressure response to CPAP treatment in subjects with obstructive sleep apnoea: the predictive value of 24-h ambulatory blood pressure monitoring. Eur Respir J. (2017) 50:1700651. doi: 10.1183/13993003.00651-2017, 28982776

[125] Sapiña-Beltrán E Torres G Benítez I Santamaría-Martos F Durán-Cantolla J Egea C. Differential blood pressure response to continuous positive airway pressure treatment according to the circadian pattern in hypertensive patients with obstructive sleep apnoea. Eur Respir J. (2019) 54:1900098. doi: 10.1183/13993003.00098-2019, 31097515

[126] Casitas R Martínez-Cerón E Galera R Cubillos-Zapata C González-Villalba MJ Fernández-Navarro I. The effect of treatment for sleep apnoea on determinants of blood pressure control. Eur Respir J. (2017) 50:1701261. doi: 10.1183/13993003.01261-2017, 29146604

[127] Hall AB Ziadi MC Leech JA Chen SY Burwash IG Renaud J. Effects of short-term continuous positive airway pressure on myocardial sympathetic nerve function and energetics in patients with heart failure and obstructive sleep apnea: a randomized study. Circulation. (2014) 130:892–901. doi: 10.1161/circulationaha.113.005893, 24993098

[128] Usui K Bradley TD Spaak J Ryan CM Kubo T Kaneko Y. Inhibition of awake sympathetic nerve activity of heart failure patients with obstructive sleep apnea by nocturnal continuous positive airway pressure. J Am Coll Cardiol. (2005) 45:2008–11. doi: 10.1016/j.jacc.2004.12.080, 15963401

[129] Saka F Cornelissen G. Chronobiologic assessment of the effect of the DASH diet on blood pressure. J Hum Hypertens. (2021) 35:678–84. doi: 10.1038/s41371-020-00408-0 32863383

[130] Marques FZ Nelson E Chu PY Horlock D Fiedler A Ziemann M. High-Fiber diet and acetate supplementation change the gut microbiota and prevent the development of hypertension and heart failure in hypertensive mice. Circulation. (2017) 135:964–77. doi: 10.1161/circulationaha.116.024545 27927713

[131] Cryan JF Dinan TG. Mind-altering microorganisms: the impact of the gut microbiota on brain and behaviour. Nat Rev Neurosci. (2012) 13:701–12. doi: 10.1038/nrn3346, 22968153

[132] Pluznick JL. Microbial short-chain fatty acids and blood pressure regulation. Curr Hypertens Rep. (2017) 19:25. doi: 10.1007/s11906-017-0722-5, 28315048 PMC5584783

[133] Juraschek SP Woodward M Sacks FM Carey VJ Miller ER 3rd Appel LJ. Time course of change in blood pressure from sodium reduction and the DASH diet. Hypertension. (2017) 70:923–9. doi: 10.1161/hypertensionaha.117.10017, 28993451 PMC5659740

[134] Wilkinson MJ Manoogian ENC Zadourian A Lo H Fakhouri S Shoghi A. Ten-hour time-restricted eating reduces weight, blood pressure, and Atherogenic lipids in patients with metabolic syndrome. Cell Metab. (2020) 31:92–104.e5. doi: 10.1016/j.cmet.2019.11.004, 31813824 PMC6953486

[135] Sutton EF Beyl R Early KS Cefalu WT Ravussin E Peterson CM. Early time-restricted feeding improves insulin sensitivity, blood pressure, and oxidative stress even without weight loss in men with prediabetes. Cell Metab. (2018) 27:1212–1221.e3. doi: 10.1016/j.cmet.2018.04.010, 29754952 PMC5990470

[136] Manoogian ENC Zadourian A Lo HC Gutierrez NR Shoghi A Rosander A. Feasibility of time-restricted eating and impacts on cardiometabolic health in 24-h shift workers: the healthy heroes randomized control trial. Cell Metab. (2022) 34:1442–1456.e7. doi: 10.1016/j.cmet.2022.08.018, 36198291 PMC9536325

[137] Hou T Su W Duncan MJ Olga VA Guo Z Gong MC. Time-restricted feeding protects the blood pressure circadian rhythm in diabetic mice. Proc Natl Acad Sci USA. (2021) 118:e2015873118. doi: 10.1073/pnas.2015873118, 34161259 PMC8237651

[138] Hou T Chacon AN Su W Katsumata Y Guo Z Gong MC. Role of sympathetic pathway in light-phase time-restricted feeding-induced blood pressure circadian rhythm alteration. Front Nutr. (2022) 9:969345. doi: 10.3389/fnut.2022.969345, 36159491 PMC9493072

[139] Uzu T Ishikawa K Fujii T Nakamura S Inenaga T Kimura G. Sodium restriction shifts circadian rhythm of blood pressure from nondipper to dipper in essential hypertension. Circulation. (1997) 96:1859–62. 9323073 10.1161/01.cir.96.6.1859

[140] Lévi FA Okyar A Hadadi E Innominato PF Ballesta A. Circadian regulation of drug responses: toward sex-specific and personalized chronotherapy. Annu Rev Pharmacol Toxicol. (2024) 64:89–114. doi: 10.1146/annurev-pharmtox-051920-095416, 37722720

[141] Hermida RC Ayala DE Fernández JR Mojón A Crespo JJ Ríos MT. Bedtime blood pressure chronotherapy significantly improves hypertension management. Heart Fail Clin. (2017) 13:759–73. doi: 10.1016/j.hfc.2017.05.010 28865783

[142] Takeda A Toda T Fujii T Matsui N. Bedtime administration of long-acting antihypertensive drugs restores normal nocturnal blood pressure fall in nondippers with essential hypertension. Clin Exp Nephrol. (2009) 13:467–72. doi: 10.1007/s10157-009-0184-4, 19449087

[143] Zeng J Jia M Ran H Tang H Zhang Y Zhang J. Fixed-combination of amlodipine and diuretic chronotherapy in the treatment of essential hypertension: improved blood pressure control with bedtime dosing-a multicenter, open-label randomized study. Hypertens Res. (2011) 34:767–72. doi: 10.1038/hr.2011.36, 21471971

[144] Hermida RC Crespo JJ Domínguez-Sardiña M Otero A Moyá A Ríos MT. Bedtime hypertension treatment improves cardiovascular risk reduction: the Hygia chronotherapy trial. Eur Heart J. (2020) 41:4565–76. doi: 10.1093/eurheartj/ehz754, 31641769

[145] Mackenzie IS Rogers A Poulter NR Williams B Brown MJ Webb DJ. Cardiovascular outcomes in adults with hypertension with evening versus morning dosing of usual antihypertensives in the UK (TIME study): a prospective, randomised, open-label, blinded-endpoint clinical trial. Lancet (London, England). (2022) 400:1417–25. doi: 10.1016/S0140-6736(22)01786-X, 36240838 PMC9631239

[146] Osborn JW Tyshynsky R Vulchanova L. Function of renal nerves in kidney physiology and pathophysiology. Annu Rev Physiol. (2021) 83:429–50. doi: 10.1146/annurev-physiol-031620-091656, 33566672

[147] Krum H Sobotka P Mahfoud F Böhm M Esler M Schlaich M. Device-based antihypertensive therapy: therapeutic modulation of the autonomic nervous system. Circulation. (2011) 123:209–15. doi: 10.1161/circulationaha.110.971580 21242507

[148] Johns EJ Kopp UC DiBona GF. Neural control of renal function. Compr Physiol. (2011) 1:731–67. doi: 10.1002/cphy.c100043 23737201

[149] Böhm M Linz D Urban D Mahfoud F Ukena C. Renal sympathetic denervation: applications in hypertension and beyond. Nat Rev Cardiol. (2013) 10:465–76. doi: 10.1038/nrcardio.2013.89, 23774592

[150] Krum H Schlaich M Whitbourn R Sobotka PA Sadowski J Bartus K. Catheter-based renal sympathetic denervation for resistant hypertension: a multicentre safety and proof-of-principle cohort study. Lancet. (2009) 373:1275–81. doi: 10.1016/s0140-6736(09)60566-3, 19332353

[151] Mauri L Kario K Basile J Daemen J Davies J Kirtane AJ. A multinational clinical approach to assessing the effectiveness of catheter-based ultrasound renal denervation: the RADIANCE-HTN and REQUIRE clinical study designs. Am Heart J. (2018) 195:115–29. doi: 10.1016/j.ahj.2017.09.006 29224639

[152] Fengler K Rommel KP Blazek S Besler C Hartung P von Roeder M. A three-arm randomized trial of different renal denervation devices and techniques in patients with resistant hypertension (RADIOSOUND-HTN). Circulation. (2019) 139:590–600. doi: 10.1161/circulationaha.118.037654, 30586691

[153] Fengler K Rommel KP Blazek S von Roeder M Besler C Hartung P. Predictors for profound blood pressure response in patients undergoing renal sympathetic denervation. J Hypertens. (2018) 36:1578–84. doi: 10.1097/hjh.0000000000001739 29652730

[154] Fengler K Rommel KP Kriese W Kresoja KP Blazek S Obradovic D. Assessment of arterial stiffness to predict blood pressure response to renal sympathetic denervation. EuroIntervention. (2022) 18:e686–94. doi: 10.4244/eij-d-21-01036, 35244604 PMC10241279

[155] Fengler K Höllriegel R Okon T Stiermaier T Rommel KP Blazek S. Ultrasound-based renal sympathetic denervation for the treatment of therapy-resistant hypertension: a single-center experience. J Hypertens. (2017) 35:1310–7. doi: 10.1097/hjh.0000000000001301, 28441700

[156] Undrum Bergland O Larstorp ACK Lund Søraas C Høieggen A Rostrup M Norheim Kjaer V. Changes in sympathetic nervous system activity after renal denervation: results from the randomised Oslo RDN study. Blood Press. (2021) 30:154–64. doi: 10.1080/08037051.2020.1868286 33399016

[157] Dobrowolski LC Eeftinck Schattenkerk DW Krediet CTP Van Brussel PM Vogt L Bemelman FJ . Renal sympathetic nerve activity after catheter-based renal denervation. EJNMMI Res. (2018) 8:8. doi: 10.1186/s13550-018-0360-1, 29374335 PMC5786599

[158] Reiter RJ Rosales-Corral SA Tan DX Acuna-Castroviejo D Qin L Yang SF . Melatonin, a full service anti-Cancer agent: inhibition of initiation, progression and metastasis. Int J Mol Sci. (2017) 18:843. doi: 10.3390/ijms18040843, 28420185 PMC5412427

[159] Zisapel N. New perspectives on the role of melatonin in human sleep, circadian rhythms and their regulation. Br J Pharmacol. (2018) 175:3190–9. doi: 10.1111/bph.14116, 29318587 PMC6057895

[160] Hung MW Kravtsov GM Lau CF Poon AM Tipoe GL Fung ML. Melatonin ameliorates endothelial dysfunction, vascular inflammation, and systemic hypertension in rats with chronic intermittent hypoxia. J Pineal Res. (2013) 55:247–56. doi: 10.1111/jpi.12067, 23869411

[161] Xia CM Shao CH Xin L Wang YR Ding CN Wang J. Effects of melatonin on blood pressure in stress-induced hypertension in rats. Clin Exp Pharmacol Physiol. (2008) 35:1258–64. doi: 10.1111/j.1440-1681.2008.05000.x, 18637016

[162] Baker J Kimpinski K. Role of melatonin in blood pressure regulation: an adjunct anti-hypertensive agent. Clin Exp Pharmacol Physiol. (2018) 45:755–66. doi: 10.1111/1440-1681.12942, 29603319

[163] Cagnacci A Cannoletta M Renzi A Baldassari F Arangino S Volpe A. Prolonged melatonin administration decreases nocturnal blood pressure in women. Am J Hypertens. (2005) 18:1614–8. doi: 10.1016/j.amjhyper.2005.05.008 16364834

[164] Grossman E Laudon M Yalcin R Zengil H Peleg E Sharabi Y. Melatonin reduces night blood pressure in patients with nocturnal hypertension. Am J Med. (2006) 119:898–902. doi: 10.1016/j.amjmed.2006.02.002, 17000226

[165] Nishi EE Almeida VR Amaral FG Simon KA Futuro-Neto HA Pontes RB. Melatonin attenuates renal sympathetic overactivity and reactive oxygen species in the brain in neurogenic hypertension. Hypertens Res. (2019) 42:1683–91. doi: 10.1038/s41440-019-0301-z, 31316170

[166] Irmak MK Sizlan A. Essential hypertension seems to result from melatonin-induced epigenetic modifications in area postrema. Med Hypotheses. (2006) 66:1000–7. doi: 10.1016/j.mehy.2005.10.016, 16434146

[167] Kalsbeek A Garidou ML Palm IF Van Der Vliet J Simonneaux V Pévet P . Melatonin sees the light: blocking GABA-ergic transmission in the paraventricular nucleus induces daytime secretion of melatonin. Eur J Neurosci. (2000) 12:3146–54. doi: 10.1046/j.1460-9568.2000.00202.x, 10998098

[168] Patel KP Li YF Hirooka Y. Role of nitric oxide in central sympathetic outflow. Exp Biol Med (Maywood). (2001) 226:814–24. doi: 10.1177/153537020122600902 11568303

[169] Fan SM Chang YT Chen CL Wang WH Pan MK Chen WP. External light activates hair follicle stem cells through eyes via an ipRGC-SCN-sympathetic neural pathway. Proc Natl Acad Sci USA. (2018) 115:E6880–e6889. doi: 10.1073/pnas.1719548115, 29959210 PMC6055137

[170] Wang F Li J Wu C Yang J Xu F Zhao Q. The GABA(a) receptor mediates the hypnotic activity of melatonin in rats. Pharmacol Biochem Behav. (2003) 74:573–8. doi: 10.1016/s0091-3057(02)01045-6, 12543221

[171] Oishi A Cecon E Jockers R. Melatonin receptor signaling: impact of receptor oligomerization on receptor function. Int Rev Cell Mol Biol. (2018) 338:59–77. doi: 10.1016/bs.ircmb.2018.02.002 29699692

[172] Ma H Kang J Fan W He H Huang F. ROR: nuclear receptor for melatonin or not? Molecules. (2021) 26:2693. doi: 10.3390/molecules26092693, 34064466 PMC8124216

[173] Andersson U Tracey KJ. Neural reflexes in inflammation and immunity. J Exp Med. (2012) 209:1057–68. doi: 10.1084/jem.20120571, 22665702 PMC3371736

[174] Chavan SS Tracey KJ. Essential Neuroscience in Immunology. J Immunol. (2017) 198:3389–97. doi: 10.4049/jimmunol.1601613, 28416717 PMC5426063

[175] Chatterjee NA Singh JP. Novel interventional therapies to modulate the autonomic tone in heart failure. JACC Heart Fail. (2015) 3:786–802. doi: 10.1016/j.jchf.2015.05.008 26364257

[176] Deuchars SA Lall VK Clancy J Mahadi M Murray A Peers L. Mechanisms underpinning sympathetic nervous activity and its modulation using transcutaneous vagus nerve stimulation. Exp Physiol. (2018) 103:326–31. doi: 10.1113/ep086433, 29205954 PMC5887928

[177] Wang Z Yu L Wang S Huang B Liao K Saren G. Chronic intermittent low-level transcutaneous electrical stimulation of auricular branch of vagus nerve improves left ventricular remodeling in conscious dogs with healed myocardial infarction. Circ Heart Fail. (2014) 7:1014–21. doi: 10.1161/circheartfailure.114.001564, 25332149

[178] Wang Z Yu L Huang B Wang S Liao K Saren G. Low-level transcutaneous electrical stimulation of the auricular branch of vagus nerve ameliorates left ventricular remodeling and dysfunction by downregulation of matrix metalloproteinase 9 and transforming growth factor β1. J Cardiovasc Pharmacol. (2015) 65:342–8. doi: 10.1097/fjc.0000000000000201, 25502306

[179] Yu L Wang S Zhou X Wang Z Huang B Liao K. Chronic intermittent low-level stimulation of Tragus reduces cardiac autonomic remodeling and ventricular arrhythmia Inducibility in a post-infarction canine model. JACC Clin Electrophysiol. (2016) 2:330–9. doi: 10.1016/j.jacep.2015.11.006 29766893

[180] Chen M Zhou X Liu Q Sheng X Yu L Wang Z. Left-sided noninvasive Vagus nerve stimulation suppresses atrial fibrillation by upregulating atrial gap junctions in canines. J Cardiovasc Pharmacol. (2015) 66:593–9. doi: 10.1097/fjc.0000000000000309, 26317165

[181] Yu L Scherlag BJ Li S Fan Y Dyer J Male S. Low-level transcutaneous electrical stimulation of the auricular branch of the vagus nerve: a noninvasive approach to treat the initial phase of atrial fibrillation. Heart Rhythm. (2013) 10:428–35. doi: 10.1016/j.hrthm.2012.11.019, 23183191

[182] Zhou L Filiberti A Humphrey MB Fleming CD Scherlag BJ Po SS. Low-level transcutaneous vagus nerve stimulation attenuates cardiac remodelling in a rat model of heart failure with preserved ejection fraction. Exp Physiol. (2019) 104:28–38. doi: 10.1113/ep087351, 30398289 PMC6312463

[183] Liu S Yu X Luo D Qin Z Wang X He W. Ablation of the ligament of Marshall and left stellate ganglion similarly reduces ventricular arrhythmias during acute myocardial infarction. Circ Arrhythm Electrophysiol. (2018) 11:e005945. doi: 10.1161/circep.117.005945, 29700056

[184] Yu L Wang M Hu D Huang B Zhou L Zhou X. Blocking the Nav1.8 channel in the left stellate ganglion suppresses ventricular arrhythmia induced by acute ischemia in a canine model. Sci Rep. (2017) 7:534. doi: 10.1038/s41598-017-00642-6, 28373696 PMC5428783

[185] Yu L Zhou L Cao G Po SS Huang B Zhou X. Optogenetic modulation of cardiac sympathetic nerve activity to prevent ventricular arrhythmias. J Am Coll Cardiol. (2017) 70:2778–90. doi: 10.1016/j.jacc.2017.09.1107 29191327

